# CCN2 (Cellular Communication Network factor 2) in the bone marrow microenvironment, normal and malignant hematopoiesis

**DOI:** 10.1007/s12079-020-00602-2

**Published:** 2021-01-11

**Authors:** Roos J. Leguit, Reinier A. P. Raymakers, Konnie M. Hebeda, Roel Goldschmeding

**Affiliations:** 1 Department of Pathology, University Medical Center Utrecht, H04-312, P.O. Box 85500, 3508 GA Utrecht, The Netherlands; 2 Department of Hematology, UMCU Cancer Center, Heidelberglaan 100 B02.226, 3584 CX Utrecht, The Netherlands; 3 Department of Pathology, Radboud University Medical Centre, P.O. Box 9101, 6500 HB Nijmegen, The Netherlands; 4 Department of Pathology, University Medical Centre Utrecht, P.O. Box 85500, 3508 GA Utrecht, The Netherlands

**Keywords:** CCN2, CTGF, Connective tissue growth factor, Bone marrow, Hematopoiesis, Leukemogenesis

## Abstract

CCN2, formerly termed Connective Tissue Growth Factor, is a protein belonging to the Cellular Communication Network (CCN)-family of secreted extracellular matrix-associated proteins. As a matricellular protein it is mainly considered to be active as a modifier of signaling activity of several different signaling pathways and as an orchestrator of their cross-talk. Furthermore, CCN2 and its fragments have been implicated in the regulation of a multitude of biological processes, including cell proliferation, differentiation, adhesion, migration, cell survival, apoptosis and the production of extracellular matrix products, as well as in more complex processes such as embryonic development, angiogenesis, chondrogenesis, osteogenesis, fibrosis, mechanotransduction and inflammation. Its function is complex and context dependent, depending on cell type, state of differentiation and microenvironmental context. CCN2 plays a role in many diseases, especially those associated with fibrosis, but has also been implicated in many different forms of cancer. In the bone marrow (BM), CCN2 is highly expressed in mesenchymal stem/stromal cells (MSCs). CCN2 is important for MSC function, supporting its proliferation, migration and differentiation. In addition, stromal CCN2 supports the maintenance and longtime survival of hematopoietic stem cells, and in the presence of interleukin 7, stimulates the differentiation of pro-B lymphocytes into pre-B lymphocytes. Overexpression of CCN2 is seen in the majority of B-acute lymphoblastic leukemias, especially in certain cytogenetic subgroups associated with poor outcome. In acute myeloid leukemia, CCN2 expression is increased in MSCs, which has been associated with leukemic engraftment in vivo. In this review, the complex function of CCN2 in the BM microenvironment and in normal as well as malignant hematopoiesis is discussed. In addition, an overview is given of data on the remaining CCN family members regarding normal and malignant hematopoiesis, having many similarities and some differences in their function.

## Introduction

### The bone marrow microenvironment

The bone marrow (BM) is the most important source of blood cells in the adult. It is the primary site where hematopoietic stem cells (HSCs) reside and give rise to more restricted progenitor cells that mature to become the different specialized blood cells. Besides hematopoietic cells, the BM contains mesenchymal stem cells, various more mature mesenchymal cells such as fibroblasts, adipocytes, osteoblasts, endothelial cells and perivascular stromal cells, as well neuronal cells and extracellular matrix (ECM), which together constitute the BM microenvironment. Integrins and selectins on the cell surface of stromal and hematopoietic cells mediate cell–cell and cell–matrix interactions (Anthony and Link 2014; Lindner et al. 2010).

One of the most important functions of the BM microenvironment is the formation of so called ‘niches’, local tissue microenvironments for the maintenance, regulation and differentiation of the HSCs (the stem cell niche) and other hematopoietic cells (Morrison and Spradling 2008). Different niches are recognized in the BM; the osteoblastic (endosteal) niche localized at the inner surface of the bone cavity where calcium levels are high, the (peri)vascular niches subdivided into a (peri)sinusoidal niche and a (peri)arteriolar niche containing high levels of stem cell factor (SCF) and CXC chemokine ligand-12 (CXCL12), the erythroid niche with a central macrophage, and a niche for lymphopoiesis located in the perisinusoidal region where both CXCL12 and interleukin 7 (IL-7) levels are high (Acar et al. 2015; Asada et al. 2017; Aurrand-Lions and Mancini 2018; Chasis and Mohandas 2008; Fujita et al. 2015; Ho and Méndez-Ferrer 2020; Lazzari and Butler 2018; Morrison and Scadden 2014; Pinho and Frenette 2019; Wei and Frenette 2018). Actively cycling, short-term HSCs as well as quiescent long-term HSCs are mainly localized in close proximity to vascular niches, but the different roles of the two vascular niches, arteriolar and sinusoidal, in HSC function are still not fully understood (Acar et al. 2015; Asada et al. 2017; Aurrand-Lions and Mancini 2018; Ho and Méndez-Ferrer 2020; Lazzari and Butler 2018; Wei and Frenette 2018). Mesenchymal cells, which include mesenchymal stem/progenitor cells and more differentiated mesenchymal cells such as perivascular stromal cells and osteoblasts, are important players in these niches as they secrete cytokines and other factors to support and regulate hematopoiesis (Morrison and Scadden 2014). When changed, the BM microenvironment can also support malignant hematopoiesis. Malignant niches differ from normal niches by the interaction with malignant cells. A role for malignant niche characteristics have been implicated in both myelodysplastic and myeloproliferative neoplasms as well as in lymphoid and myeloid leukemias (Arranz et al. 2014; Balderman et al. 2016; Battula et al. 2013; Blau et al. 2007; Boyerinas et al. 2013; Guerrouahen et al. 2011; Korn and Mendez-Ferrer 2017; Lazzari and Butler 2018; Li and Calvi 2017; Sangaletti et al. 2017; Schepers et al. 2013).

Homeostasis of the BM microenvironment is tightly regulated by a complex and not fully elucidated interplay between different cell types, structural ECM components and soluble factors such as cytokines, hormones, growth factors and matricellular proteins. Matricellular proteins are proteins secreted into the extracellular environment, modulating cell function and cell–matrix interactions by binding to cell-surface receptors, structural matrix proteins and other soluble matrix proteins (Bornstein 2009; Bornstein and Sage 2002). The Cellular Communication Network (CCN) family forms an important group of matricellular proteins (Chen and Lau 2009). Bork was the first to conceive the different CCN proteins as members of a (single) family (Bork 1993), that now consists of six structural related proteins.

The CCN proteins act as central mediators of mechanotransduction and play important roles in, amongst others, angiogenesis, inflammation, connective tissue deposition, and a broad range of pathological processes including fibrosis and cancer (Chaqour 2020; Leask 2020). The proteins of the CCN family might play as a team and ideally should be assessed together rather than individually (Peidl et al. 2019; Perbal 2018; Riser et al. 2009, 2010), but for most of them, little is known about their possible involvement in hematopoiesis and the BM microenvironment. Since CCN2 is by far the most studied CCN protein in this field, it will therefore be the focus of this review, and the more limited available knowledge on the other CCN proteins is included at the end.

### CCN2 protein

Although CCN2 was originally thought to be a classical growth factor and named connective tissue growth factor (CTGF), no high-affinity classical growth factor receptor for CCN2 has been discovered (Lau 2016). Instead, CCN2 has the capacity to interact with a range of cell surface receptors, ECM macromolecules, growth factors and proteases, thereby directly or indirectly regulating cellular function, which are features common to all matricellular proteins (Bornstein 1995; Murphy-Ullrich and Sage 2014). Furthermore, all four structural domains of the CCN proteins are homologous to other ECM-associated proteins (Lau and Lam 1999). Therefore, the protein originally called CTGF is nowadays considered a matricellular protein and has been renamed CCN2 by the HUGO Gene Nomenclature Committee (Brigstock et al. 2003; Perbal et al. 2018).

**Fig. 1:**
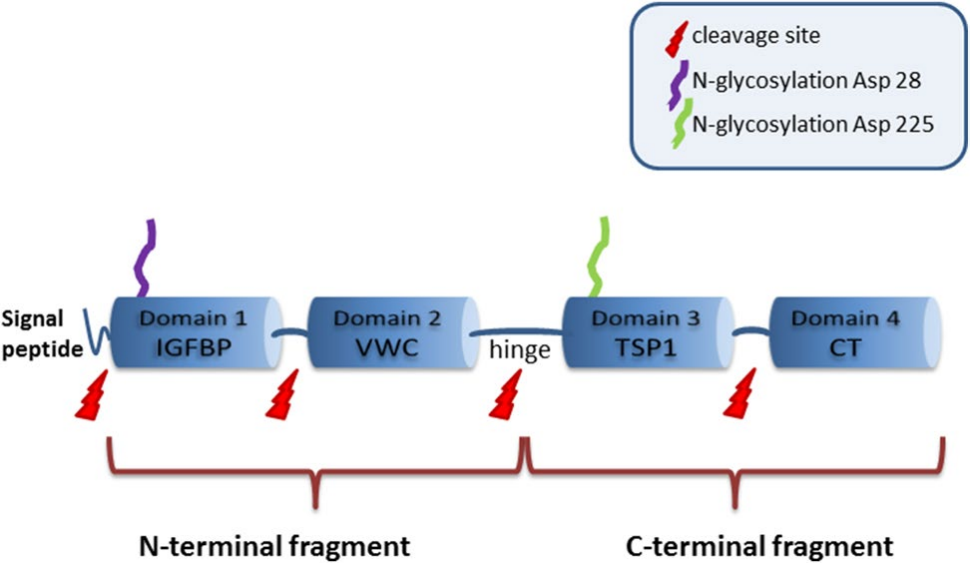
CCN2 protein structure. Schematic representation of the full length CCN2 protein, which is made up by a signal peptide and 4 protein domains, depicted by the blue cylinders. Domain 1 consists of the insulin-like growth factor binding protein (IGFBP) module and domain 2 contains the von Willebrand factor C (VWC) module, together forming the N-terminal fragment of the protein. Domain 3 consists of the thrombospondin type 1 repeat (TSP1) module and domain 4 contains the C-terminal cysteine knot (CT) module, together forming the C-terminal fragment of the protein. The N- and C-terminal fragments are joint by a hinge region. Between the different domains, multiple cleavage sites are present, were CCN2 is cleaved by proteases, plasmin, chymotrypsin and matrix metalloproteinases. Loss of the signal peptide leads to intracellular retention of the protein. The protein contains 2 glycosylation sites. The functional relevance of glycosylation is, however, still unknown. Other (not depicted) posttranscriptional and posttranslational modifications to which the protein is subject to, are splicing, regulation by miRNAs and multimerisation

CCN2 (Fig. 1) is a cysteine-rich 36–38 kDa (depending on the level of N-linked glycosylation) protein (Bradham et al. 1991; Yang et al. 1998), consisting of a secretory signal peptide and four functionally distinct and highly conserved domains/modules (Bork 1993; Holbourn et al. 2008). The N-terminal fragment of CCN2 is made up by an insulin-like growth factor binding protein (IGFBP) module and a von Willebrand factor C (VWC) module (Brigstock 1999; Lau and Lam 1999), which is linked by a hinge region to the C-terminal fragment consisting of a thrombospondin type 1 repeat (TSP1) module and a C-terminal cysteine knot (CT) module (Brigstock 1999; Lau and Lam 1999). Each module interacts with other extracellular proteins and/or proteoglycans (Perbal 2018). CCN2 is mainly secreted as an extracellular protein and has been identified in various human biological fluids (Bradham et al. 1991; Yang et al. 1998). Loss of its 2 kDa N-terminal secretory signal peptide leads to intracellular retention of the protein (Welch et al. 2015).

The cysteine-free hinge region between modules 2 and 3 contains multiple cleavage sites susceptible to proteolysis by a number of different proteases (de Winter et al. 2008; Grotendorst and Duncan 2005; Hashimoto et al. 2002). In addition, CCN2 can be cleaved between module 1 and 2 and between module 3 and 4 into fragments that represent the individual motifs (de Winter et al. 2008; Grotendorst and Duncan 2005; Hashimoto et al. 2002). The different modules and fragments have been associated with specific biological roles (Abd El Kader et al. 2014; Ball et al. 2003; de Winter et al. 2008; Grotendorst and Duncan 2005; Johnson et al. 2017; Kubota et al. 2006; Mokalled et al. 2016; Tong and Brigstock 2006). As truncated proteins missing one or more modules have even been reported to possess *enhanced* biological activities, CCN2 has been suggested to be a prepro-protein that may undergo proteolytic cleavage in order to release biologically more active fragments (Abd El Kader et al. 2014; Kaasboll et al. 2018). On the other hand, some of the biological activities of CCN2 require the intact, full length protein with the individual modules acting together (Kubota et al. 2006). Studying CCN2, however, remains challenging as it is difficult to purify larger amounts of native, biologically active CCN proteins in a stable form as pointed out by Perbal (2018).

### CCN2 function and expression

CCN2 is expressed in a variety of tissues during embryonic development, with highest levels in vascular tissues and maturing chondrocytes (Hall-Glenn et al. 2012; Ivkovic et al. 2003). In the adult, CCN2 expression can be induced in various cell types, including endothelial cells (Bradham et al. 1991; Lee et al. 2015; Yan et al. 2014), vascular smooth muscle cells (Gao et al. 2007; Ko et al. 2012; Liu et al. 2008; Rodriguez-Vita et al. 2005), chondrocytes (Nakanishi et al. 1997), fibroblasts (Grotendorst 1997; Guo et al. 2011; Holmes et al. 2003; Igarashi et al. 1993) and mesangial cells (Goppelt-Struebe et al. 2001). CCN2 acts in an autocrine or paracrine fashion and its regulation and modes of action are complex and context dependent, depending on cell type, state of differentiation, and microenvironmental context (Cicha and Goppelt-Struebe 2009; Guo et al. 2011).

As previously reviewed, CCN2 and its fragments have been implicated in the regulation of a multitude of biological phenomena, including cell proliferation, differentiation, adhesion, migration, cell survival, apoptosis and the production of ECM products (de Winter et al. 2008; Jun and Lau 2011; Takigawa 2018), as well as in embryonic development, angiogenesis, chondrogenesis, osteogenesis, fibrosis, mechanotransduction and inflammation (Chaqour 2020; Jun and Lau 2011; Kubota and Takigawa 2013; Takigawa 2013, 2018). It should be noted, however, that at least several of these propositions have not been based on robust assays using sufficiently characterized and purified CCN2 and its fragments. As discussed by Leask, adhesion assays are probably the only robust, universally agreed-upon in vitro assays for assessing CCN activity, at least of full-length CCN proteins (Leask 2020).

**Fig. 2:**
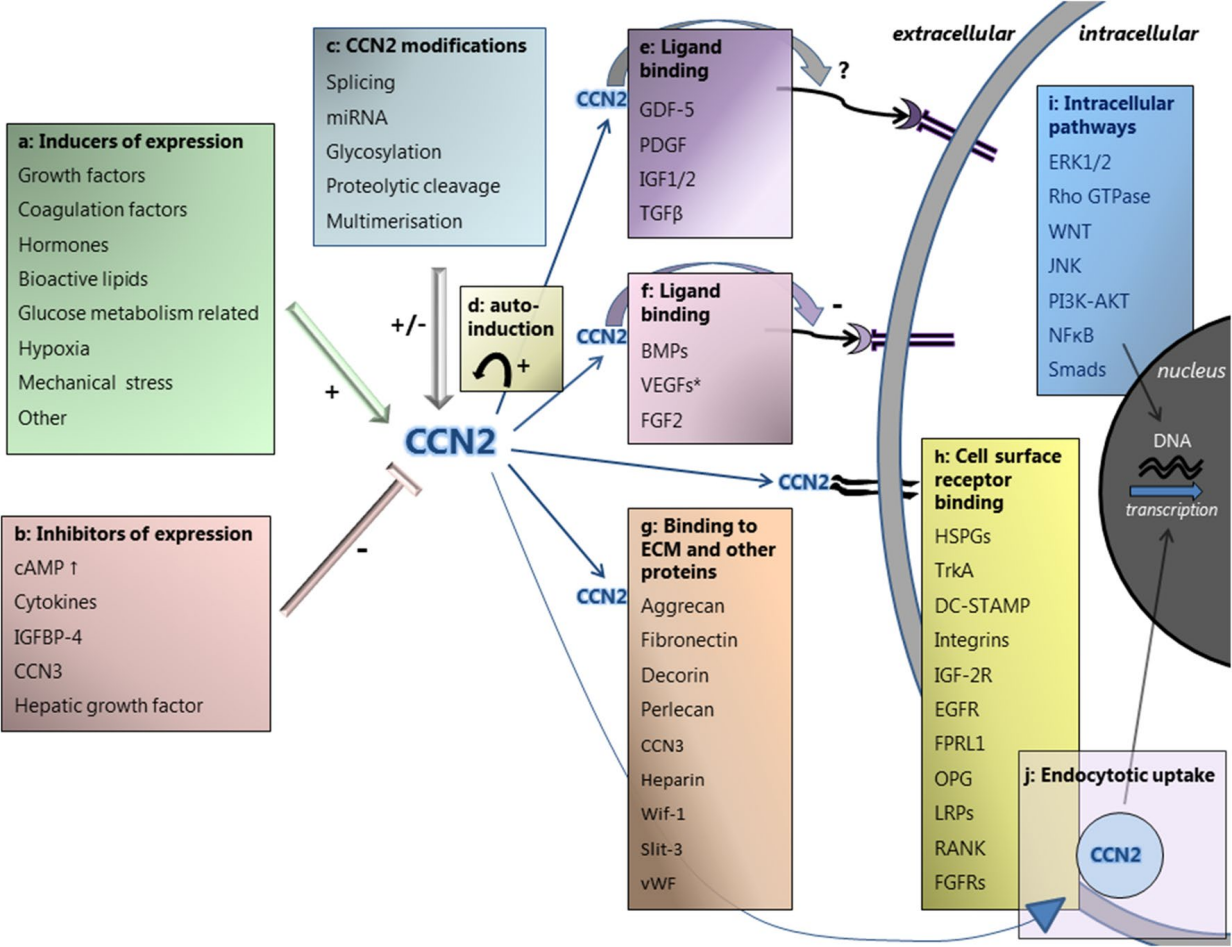
Overview of the complex regulation of CCN2 expression, and the diverse actions of the CCN2 protein, as reported in literature. *Regulation of CCN2 expression.* **a** Factors inducing CCN2 expression include growth factors, coagulation factors, hormones, bioactive lipids, glucose metabolism related factors, hypoxia and mechanical stress. **b** Factors inhibiting CCN2 expression include increased levels of cyclic adenosine monophosphate (cAMP), cytokines, insulin‐like growth factor binding protein‐4 (IGFBP-4), CCN3 and hepatic growth factor. **c** CCN2 expression is modified by posttranscriptional and posttranslational factors, which includes splicing, regulation by miRNAs, glycosylation, proteolytic cleavage and multimerisation of the protein. **d** CCN2 can induce its own expression by auto-induction, resulting in a positive feedback loop. *Actions of the CCN2 protein:* CCN2 exerts its function by binding to growth factors and cell surface receptors, thereby affecting intracellular signaling, as well as by binding to extracellular matrix (ECM) and other proteins. **e** CCN2 binds to growth-differentiation factor 5 (GDF-5), platelet derived growth factor (PDGF), insulin-like growth factor-1 and 2 (IGF1/2) and transforming growth factor-β (TGF-β). The effect of CCN2 on the binding of these ligands to their putative receptors is not fully elucidated. **f** CCN2 binds to bone morphogenetic proteins (BMPs), vascular endothelial growth factors (VEGFs) and fibroblast growth factor 2 (FGF2), thereby modulating their presentation and binding to cell-surface receptors, resulting in inhibited signal transduction. *Only full-length CCN2 inhibits VEGFA and VEGFC, not its proteolytic fragments. **g** CCN2 binds directly to structural ECM proteins, matricellular proteins and other proteins, including aggrecan, fibronectin, decorin, perlecan, CCN3, heparin, Wnt inhibitory factor 1 (Wif-1), Slit guidance ligand 3 (Slit-3), and von Willebrand factor (vWF). **h** CCN2 can bind directly to cell surface receptors, which include heparan sulphate proteoglycans (HSPGs), tropomyosin receptor kinase A (TrkA), dendritic cell-specific transmembrane protein (DC-STAMP), integrins, insulin-like growth factor 2 receptor (IGF-2R), epidermal growth factor receptor (EGFR), formyl peptide receptor-like 1 (FPRL1), osteoprotegerin (OPG), lipoprotein receptor-related proteins (LRPs), receptor activator of NF-κB (RANK), and fibroblast growth factor receptors (FGFRs). By CCN2 binding, intracellular signaling pathways and their cross-talk can be altered and gene transcription affected. **i** Intracellular signaling pathways that are affected by CCN2 include the extracellular signal–regulated kinase 1 and 2 (ERK1/2) pathway, the Rho GTPase pathway, the WNT pathway, the c-Jun N-terminal kinase (JNK) pathway, the phosphatidylinositol 3‑kinase (PI3K)/AKT pathway and the nuclear factor κB (NFκB) pathway the signaling mother against dexapentaplegic peptides (Smad) pathway. **j** CCN2 has been reported to act intracellularly after being taken up into the cytosol via endocytic pathways and, after phosphorylation, into the nucleus where it may affect gene transcription

CCN2 has the ability to interact with a wide variety of proteins and receptors by its different modules, and is considered to be active as a modifier of signaling activity of several different signaling pathways and as an orchestrator of their cross-talk (Leask 2020; Perbal 2018; Ramazani et al. 2018). CCN2 can regulate biological processes in various ways (Fig. 2): (1) It can bind to several cell surface receptors, thereby initiating signal transduction, (2) It can bind to growth factors, modulating their presentation and binding to cell-surface receptors, and subsequent initiation of downstream signaling pathways, (3) It has a modifying role in mediating matrix turnover by binding to (structural) ECM proteins, (4) It is involved in the regulation of the activity of cytokines and growth factors through modulation of crosstalk between signaling pathways, and (5) It has been reported to act intracellularly, after uptake into the cytosol via endocytic pathways and into the nucleus, where it may affect gene transcription (Kawata et al. 2006, 2012; Lau 2016; Ramazani et al. 2018; Takigawa 2018; Wahab et al. 2001).

The reported binding partners of CCN2 are summarized in Table 1 and depicted in Fig. 2. It should be noted here that at least some of the interactions of CCN2 with other molecules have been studied only in ex vivo conditions under circumstances that might not be fully representative of in vivo conditions. By binding to cell surface receptors, CCN2 can alter various intracellular signaling pathways, including the ERK pathway (Aoyama et al. 2012; Lee et al. 2015; Rayego-Mateos et al. 2013; Yang et al. 2004), WNT pathway (Liu and Leask 2013; Mercurio et al. 2004; Rooney et al. 2011), JNK pathway (Aoyama et al. 2015; Yosimichi et al. 2006), NFκB pathway (Aoyama et al. 2015; Edwards et al. 2011) and others (Wahab et al. 2005; Yosimichi et al. 2006). Its binding to growth factors usually has an inhibitory effect: Binding to bone morphogenetic proteins (BMPs) inhibits BMP signaling (Abreu et al. 2002; Maeda et al. 2009; Mundy et al. 2014; Nguyen et al. 2008), resulting in reduced phosphorylation of EKR and Smad 1/5/8 (Maeda et al. 2009; Mundy et al. 2014; Nguyen et al. 2008), and binding to vascular endothelial growth factor 165 (VEGF_165_) inhibits its angiogenic activity by interrupting binding to its major receptor VEGFR-2 (Heroult et al. 2004; Inoki et al. 2002). Binding of the CT module, but not full length CCN2, to fibroblast growth factor 2 (FGF2) inhibits its binding to fibroblast growth factor receptor-1 (FGFR1) and thereby its activation (Nishida et al. 2011b). Furthermore, there are indications that CCN2 has an inhibitory effect on insulin-like growth factor (IGF) signaling and WNT signaling (Smerdel-Ramoya et al. 2008). Binding of CCN2 to transforming growth factor beta (TGF-β) has been reported to enhance TGF-β signaling (Abreu et al. 2002). This interesting notion has, however, not been reproduced in more recent literature.

**Table 1:** Reported binding partners of CCN2

Factor	Abbreviation	References
*Cell surface receptors*		
Integrins		Babic et al. (1999), Ball et al. (2003), Chen et al. (2001), Gao and Brigstock (2004), Gao and Brigstock (2005), Jedsadayanmata et al. (1999), Kiwanuka et al. (2013), Lau (2016), Liu et al. (2012), Rayego-Mateos et al. (2013), Schober et al. (2002)
Lipoprotein receptor-related protein-1 -4, and -6	LRP-1, LRP-4, LRP-6	Gao (2003), Kawata et al. (2012), Mercurio et al. (2004), Ohkawara et al. (2020), Ren et al. 2013; Rooney et al. (2011), Segarini et al. (2001), Yang et al. (2004)
Neurotrophin receptors: Tropomyosin receptor kinase A and P75^NTR^	TrkA, P75^NTR^	Edwards et al. (2011), Rayego-Mateos et al. (2013), Wahab et al. (2005), Wang et al. (2010)
Insulin-like growth factor 2 receptor/Cation-independent mannose-6-phosphate receptor	IGF-2-R/M6P	Blalock et al. (2012)
Fibroblast growth factor receptor 1, 2 and 3	FGFR-1, FGFR-2, FGFR-3	Aoyama et al. (2012), Nishida et al. (2011b)
Epidermal growth factor receptor	EGFR	Rayego-Mateos et al. (2013)
Receptor activator of NF-κB	RANK	Aoyama et al. (2015)
Dendritic cell-specific transmembrane protein	DC-STAMP	Nishida et al. (2011a)
Osteoprotegerin	OPG	Aoyama et al. (2015)
Formyl peptide receptor-like 1	FPRL1	Lee et al. (2015)
*Growth factors*		
Transforming growth factor beta	TGF-β	Abreu et al. (2002) and Khattab et al. (2015)
Bone morphogenetic protein 2, 4 and 7	BMP-2, BMP-4, MBP-7	Abreu et al. (2002), Maeda et al. (2009), Nguyen et al. (2008)
Platelet derived growth factor-B and -BB	PDGF-BB, PDGF-B	Khattab et al. (2015), Pi et al. (2012)
Vascular endothelial growth factors-A and -C	VEGF-A, VEGF-C	Heroult et al. (2004), Inoki et al. (2002), Khattab et al. (2015), Kinashi et al. (2017)
Fibroblast growth factor 2	FGF2	Nishida et al. (2011b)
Insulin-like growth factors 1 and 2	IGF1/2	Kim et al. (1997), Lam et al. (2003)
Growth-differentiation factor 5	GDF-5	Khattab et al. (2015)
*Structural matrix proteins*		
Perlecan		Nishida et al. (2003)
Aggrecan		Aoyama et al. (2009)
Fibronectin		Hoshijima et al. (2006), Pi et al. (2008)
Decorin		Vial et al. (2011)
*Other*		
Heparin		Ball et al. (2003), Brigstock et al. (1997), Frazier et al. (1996), Gao (2003), Kireeva et al. (1997)
Heparan sulphate proteoglycans (cell surface and matrix associated)	HSPGs	Ball et al. (2003), Chen et al. (2001), Gao (2003), Gao and Brigstock (2004), Kireeva et al. (1997), Nishida et al. (2003)
CCN2		Hoshijima et al. (2012)
CCN3		Hoshijima et al. (2012)
Wnt inhibitory factor 1 (Wif-1)	Wif-1	Surmann-Schmitt et al. (2012)
Slit guidance ligand 3	Slit-3	Pi et al. (2012)
von Willebrand factor	vWF	Pi et al. (2012)

In addition to its direct effects on cell surface receptors and growth factors, CCN2 can increase the level of matrix metalloproteinases (MMPs), a large family of enzymes playing a central role in the ECM by the degradation of specific ECM components and cleavage of growth factors and their binding proteins, by upregulating their gene expression in fibroblasts and endothelial cells (Chen et al. 2001; Fan and Karnovsky 2002; Kondo et al. 2002). On the other hand, CCN2 itself is a substrate of several MMPs, by which it can be cleaved in the hinge region (Dean et al. 2007; Hashimoto et al. 2002; Tam et al. 2004). For an overview and more detail of CCN2 binding partners and intracellular signaling pathways we like to refer to several recent reviews (Chaqour 2020; Lau 2016; Ramazani et al. 2018; Takigawa 2018).

### Regulation of CCN2 expression

TGF-β is a powerful and well-known inducer of CCN2 transcription (Brunner et al. 1991; Grotendorst et al. 1996; Holmes et al. 2001; Igarashi et al. 1993; Yang et al. 1998), but many other factors, summarized in Table 2 and depicted in Fig. 2, can also directly or indirectly induce CCN2 mRNA expression through the initiation of signaling pathways and the activation of transcription factors, as previously reviewed (Chaqour 2020; Jun and Lau 2011; Ramazani et al. 2018; Takigawa 2018). For example, on mechanical stress, the transcriptional regulators YAP (Yes-associated protein) and TAZ (transcriptional coactivator with PDZ-binding motif) induce CCN2 gene transcription (Dupont et al. 2011; Nagasawa-Masuda and Terai 2017; Preisser et al. 2016; Raghunathan et al. 2014). FAK (focal adhesion factor) is an important factor in mechanotransduction, which controls the nuclear translocation and activation of YAP and subsequent CCN2 gene expression in response to mechanical activation (Lachowski et al. 2018). In addition, CCN2 can induce its own expression by auto-induction, resulting in a positive feedback loop (Riser et al. 2000; Shimo et al. 2001b). Other factors, summarized in Table 3 and depicted in Fig. 2, can directly or indirectly inhibit CCN2 expression. For involvement of specific regulatory elements in the CCN2 gene we refer to Leask et al. (2009). Furthermore, CCN2 expression can be regulated at the posttranscriptional and posttranslational level by various factors, including VEGF (Kondo et al. 2006), hypoxia (Kondo et al. 2002), tumor necrosis factor α (TNF-α), interferon gamma (IFN-γ) (Cooker et al. 2007; Laug et al. 2012), and a host of different microRNAs (miRNAs) (Cai et al. 2018; Che et al. 2019; Chen et al. 2016, 2019; Ernst et al. 2010; Fox et al. 2013; He et al. 2017; Mu et al. 2016; Qiao et al. 2017; Sun et al. 2016). For example, CCN2 and miRNA-21 are components of a positive feedback loop in pancreatic stellate cells, that may serve as an amplification mechanism for enhanced collagen production (Charrier et al. 2014a). On the other hand, CCN2 can increase the expression of miRNA-302, which targets the TGFβ type II receptor and thereby decreases its expression, constituting a negative feedback loop (Faherty et al. 2012).

**Table 2:** Reported (direct or indirect) inducers of CCN2 mRNA expression

Factor	References
*Growth factors*	
TGF-β	Gao and Brigstock (2005), Goppelt-Struebe et al. (2001), Grotendorst et al. (1996), Holmes et al. (2003), Igarashi et al. (1993), Nakanishi et al. (1997), Riser et al. (2000), Wunderlich et al. (2000)
PDGF	Gao and Brigstock (2005)
Epidermal growth factor (EGF)	Wunderlich et al. (2000)
Basic fibroblast growth factor (bFGF)	Wunderlich et al. (2000)
BMP-2	Nakanishi et al. (1997)
VEGF	Lee et al. (2015), Suzuma et al. (2000)
*Hormones*	
Steroids	Dammeier et al. (1998), Kubota et al. (2003), Moritani et al. (2005), Okada et al. (2006), Pereira et al. (2000)
Angiotensin II	Gao et al. (2007), Gu et al. (2012)
Endothelin-1	Kemp et al. (2004), Rodriguez-Vita et al. (2005), Shi-Wen et al. (2007)
Aldosteron	Lee et al. (2004)
*Coagulation factors*	
Thrombin	Bai et al. (2013), Chambers et al. (2000)
Factor Xa	Chambers et al. (2000)
*Glucose metabolism related*	
Glucose	Lam et al. (2003), Murphy et al. (1999), Paradis et al. (2001), Riser et al. (2000)
Glycolysis—via adenosine triphosphate (ATP)	Akashi et al. (2018)
Advanced glycosylation end products	Twigg et al. (2001)
Insulin	Paradis et al. (2001)
*Cytokines*	
Tumor necrosis factor alpha (TNF-α)^a^	Cooker et al. (2007)
*Other*	
Bioactive lipids	Chowdhury and Chaqour (2004), Goppelt-Struebe et al. (2001), Muehlich et al. (2004)
Ethanol and acetaldehyde	Charrier et al. (2014b), Gao and Brigstock (2005)
UV-light	Kafi et al. (2004), Quan et al. (2009)
Mechanical stress (shear and cell stretch)	Guo et al. (2011), Honjo et al. (2012), Kessler et al. (2001), Riser et al. (2000), Schild and Trueb (2002), Schild and Trueb (2004), Wong et al. (2003), Yamashiro et al. (2001)
Hypoxia—via hypoxia-inducible-factor-1 (HIF-1α)	Higgins et al. (2004), Kondo et al. (2002), Shimo et al. (2001a), Valle-Tenney et al. (2020)
Nitric oxide (NO)	Tsai et al. (2014)
CCN2	Bradham et al. (1991), Kawaki et al. (2008), Parada et al. (2013), Riser et al. (2000), Shimo et al. (2001b)
Insulin-like growth factor-binding protein 5 (IGFBP-5)	Nguyen et al. (2018)

^a^TNF-α has also been reported to inhibit CCN2 mRNA expression (see Table 3)

**Table 3:** Reported (direct or indirect) inhibitors of CCN2 mRNA expression

Factor	References
*Growth factors*	
Hepatocyte growth factor	Inoue et al. (2003), Kroening et al. (2009)
*Cytokines*	
Tumor necrosis factor alpha (TNF-α)^a^	Abraham et al. (2000), Dammeier et al. (1998), Laug et al. (2012), Lin et al. (1998), Moritani et al. (2005)
Interferon-gamma (IFN-γ)	Laug et al. (2012)
Interleukin 1 beta (IL-1β)	Masuko et al. (2010)
*Elevators of cyclic adenosine monophosphate (cAMP) levels*	
Cholera toxin	Duncan et al. (1999), Kothapalli et al. (1998), Masuko et al. (2010)
Prostaglandin E2	Masuko et al. (2010), Ricupero et al. (1999)
*Other*	
CCN3	Kawaki et al. (2008)
Insulin‐like growth factor binding protein‐4 (IGFBP-4)	Su et al. (2019)

^a^TNF-α has also been reported to induce CCN2 mRNA expression (see Table 2)

### CCN2 and disease

CCN2 has been implicated in the pathophysiology of many diseases; increased CCN2 expression has been demonstrated in the tissue of a range of diseases that are accompanied by fibrosis such as fibrotic lung diseases, scleroderma, chronic pancreatitis, renal fibrosis, liver cirrhosis, myocardial infarction, and Crohn’s disease (Abou-Shady et al. 2000; di Mola et al. 1999, 2004; Ito et al. 1998; Ohnishi et al. 1998; Pan et al. 2001; Shi-wen et al. 2000), as well as in diabetic retinopathy and in the osteoarthritic cartilage of patients with osteoarthritis (Omoto et al. 2004; Tikellis et al. 2004). In addition, increased levels of CCN2 cleavage products have been demonstrated in human extracellular fluids, including plasma, urine, dermal interstitial fluid and vitreous fluid, of patients with fibrotic diseases, correlating with the severity of fibrosis (Leask et al. 2009). Furthermore, increased CCN2 plasma levels have been associated with cardiac dysfunction and increased risk of cardiovascular events in patients with atherosclerotic disease (Behnes et al. 2014; Gerritsen et al. 2016; Koitabashi et al. 2008), and altered CCN2 expression has been demonstrated in more than 25 different forms of cancer, with deregulation of CCN2 expression usually correlating with worse clinical outcome (Wells et al. 2015). Besides altered CCN2 expression in tumor cells, elevated CCN2 expression in stromal fibroblasts is implicated in the desmoplastic response in various cancer types, and CTGF expression in stromal cells can advance tumor growth or promote invasion as reviewed by Wells et al. (2015).

## CCN2 and the normal BM microenvironment (Fig. 3)

### CCN2 expression and function in mesenchymal stem and stromal cells

Mesenchymal stem cells are essential for the maintenance of the BM microenvironment; they can self-renew and have the capacity to differentiate into other mesenchymal cell types, including chondrocytes, adipocytes, fibroblasts and osteoblasts. They also stimulate the production of the ECM (Huang et al. 2016), and maintain hematopoiesis by the secretion of cytokines that stimulate proliferation of hematopoietic progenitor cells (Huang et al. 2016). Mesenchymal stem cells are rare, constituting only 1 in 3.4 × 10^4^ nucleated BM cells (Wexler et al. 2003). As it is difficult to identify mesenchymal stem cells on a per cell basis, mesenchymal stem cell-enriched cell populations of both true mesenchymal stem cells and more differentiated mesenchymal stromal cells are used in most experimental settings (Lindner et al. 2010). The mesenchymal stem and stromal cells from the cited studies are therefore further referred to as mesenchymal stem/stromal cells (MSCs).

**Fig. 3:**
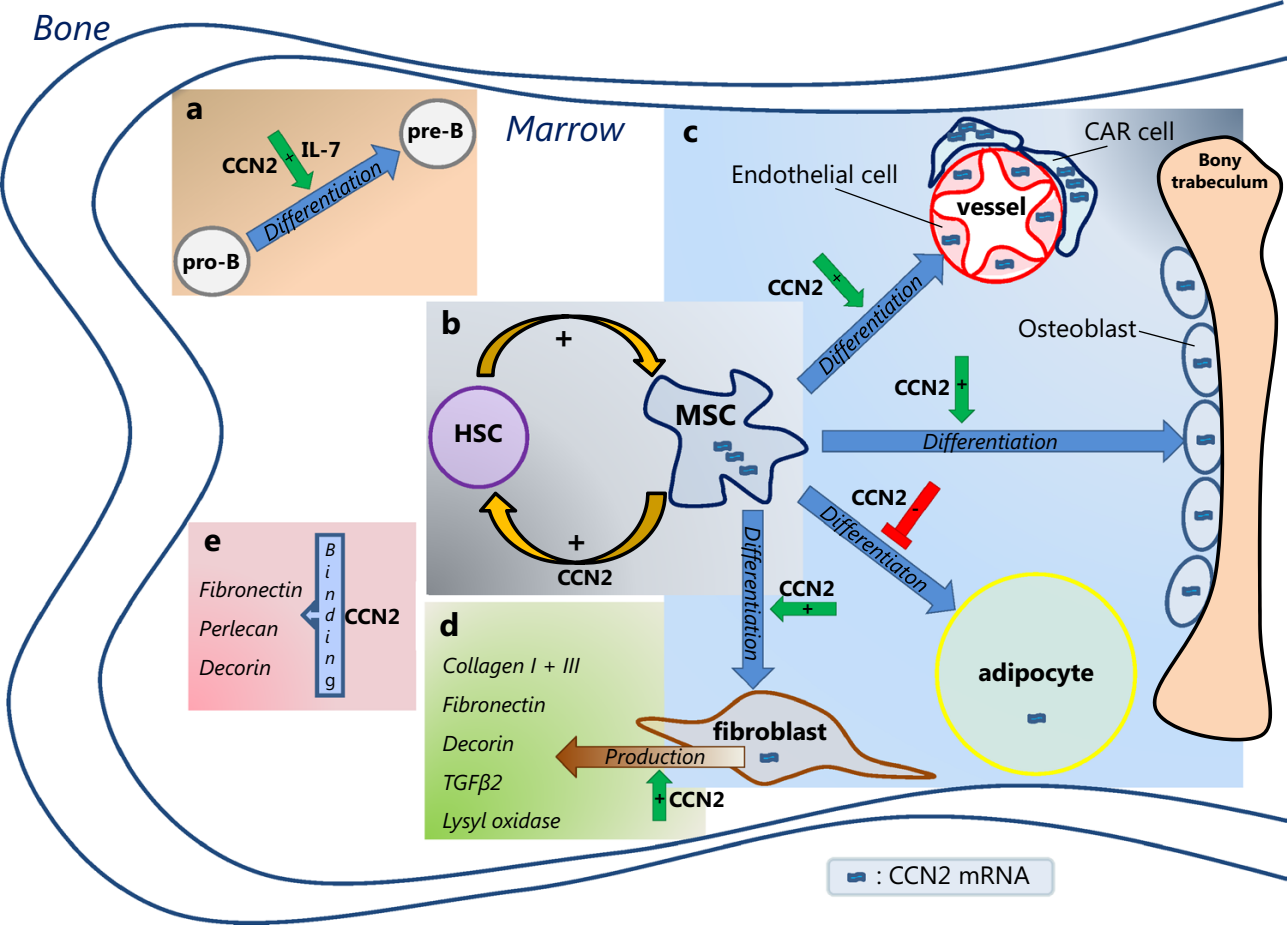
CCN2 in the bone marrow microenvironment. CCN2 mRNA, depicted by  , is present in different bone marrow (BM) mesenchymal cells, including endothelial cells, osteoblasts, adipocytes and fibroblasts, with highest levels ( ) reported in mesenchymal stem/stromal cell (MSC) and CXCL12-abundant reticular (CAR) cells. CCN2 exerts different actions in the BM. **a** In the presence of interleukin 7 (IL-7), CCN2 promotes pro-B cell to pre-B cell differentiation. **b** CCN2 produced by MSCs affects the (long-term) qualities of hematopoietic stem cells (HSCs). HSCs, in turn, upregulate CCN2 expression by MSCs. **c** CCN2 enhances the differentiation of MSCs into endothelial cells, osteoblast and fibroblasts, but has an inhibitory effect on the differentiation of MSCs into adipocytes. **d** CCN2 might induce the production of the ECM proteins collagen type I and type III, fibronectin, decorin, TGFβ-2 and lysyl oxidase by fibroblast. **e** CCN2 binds to fibronectin, perlecan and decorin, known constituents of the BM extracellular matrix. The effects hereof in the BM are yet unknown.

BM-derived MSCs show high expression of CCN2 mRNA (Battula et al. 2013, 2017; Cheung et al. 2014; Djouad et al. 2007; Igarashi et al. 2007; Ren et al. 2011; Schutze et al. 2005; Shinde et al. 2014), which can be induced in vitro by Wnt3a and BMPs (Luo et al. 2004). CCN2 is important for MSC function as indicated by several in vitro and mouse studies. Although cell numbers and proportions of MSCs isolated from the BM of newborn CCN2-knockout mice were unchanged compared with those from wild-type (WT) mice (Cheung et al. 2014), MSCs from tissues of CCN2 homozygous knockout mice were not able to generate colony-forming unit fibroblast-like cells in vitro (Battula et al. 2013), implying a functional defect. Indeed, a role for CCN2 in MSC cell growth has been demonstrated, as CCN2 is shown to enhance proliferation and inhibit apoptosis of MSCs in vitro (Battula et al. 2013; Wang et al. 2009; Wells et al. 2016). In addition, CCN2 enhances MSC cell migration and recruitment, shown by either the addition of exogenous CCN2 or through plasmid induced expression of CCN2 (Luo et al. 2004; Wang et al. 2009). Furthermore, CCN2 affects MSC differentiation. Although knockdown of endogenous CCN2 expression in MSCs did not affect their capacity to differentiate into osteoblasts or chondrocytes (Battula et al. 2013), increase of CCN2 by either exposure to recombinant CCN2 (rCCN2) or transfection of CCN2-expressing plasmids did enhance the differentiation of human BM MSCs into osteoblasts and chondrocytes as well as fibroblasts in culture (Lee et al. 2010; Nishida et al. 2004; Wang et al. 2009). MSCs can also be differentiated into myofibroblasts by the addition of rCCN2, but only when stimulated subsequently with TGF-β (Lee et al. 2010; Wang et al. 2009). On the other hand, knockdown of CCN2 expression in MSCs enhanced adipocytic differentiation (Battula et al. 2013), suggesting an inhibitory effect of CCN2 on adipogenesis. When MSCs differentiate into these progenitor cells with lineage commitment, CCN2 expression was shown to decrease (Luo et al. 2004; Schutze et al. 2005).

### CCN2 expression and function in endothelial and perivascular cells

Endothelial cells and perivascular stromal cells are part of the (peri)vascular niche and contribute to the microenvironment by the production of SCF and other growth factors, cytokines, chemokines and adhesion molecules such as E-selectin and CXCL12 (Ding and Morrison 2013; Sacchetti et al. 2007; Sipkins et al. 2005; Sugiyama et al. 2006; Winkler et al. 2012; Zhao et al. 2019).

CCN2 has been shown to induce neovascularisation and to promote the adhesion, migration, proliferation and survival of vascular endothelial cells in vitro (Babic et al. 1999; Shimo et al. 1998, 1999). CCN2 mRNA expression in endothelial cells increases in vitro by the addition of bioactive lipids such as sphingosine-1-phosphate and lysophosphatidic acid (Muehlich et al. 2004). Also, addition of freshly isolated platelets to endothelial cells upregulates their CCN2 mRNA expression (Muehlich et al. 2004), possibly due to the release of constituents of lipoproteins, TGF-β and other CCN2-inducing compounds by platelets.

As expected, CCN2 mRNA is present in endothelial cells derived from the BM as well (Cheung et al. 2014). The perivascular region of the BM contains a heterogeneous population of stromal cells characterized by very high CXCL12 expression, including the CXCL12-abundant reticular (CAR) cells, which are mesenchymal progenitor cells important for the maintenance of both HSC and B-cells (Eltoukhy et al. 2016; Sugiyama et al. 2006). These CAR-cells were shown to have the highest expression of CCN2 mRNA of all investigated BM stromal cells (Cheung et al. 2014). The cell numbers and the proportions of endothelial cells and CAR cells isolated from *CCN2*^+/−^ and *CCN2*^−/−^ newborn mice are unchanged compared with those from WT mice (Cheung et al. 2014), but their function has not been studied in these knock-out models. The role of CCN2 in the perivascular niche thus remains to be established.

### CCN2 expression and function in osteoblasts

Osteolineage cells contribute to the BM microenvironment by secreting factors such as granulocyte colony stimulating factor (G-CSF) (Taichman and Emerson 1994), thrombopoietin (TPO) (Yoshihara et al. 2007), and CXCL12 (Jung et al. 2006), although their effect on hematopoiesis is not fully determined (Ho and Méndez-Ferrer 2020). Osteolineage cells also express CCN2 (Luo et al. 2004; Safadi et al. 2003; Xu et al. 2000).

CCN2 is long known for its importance in endochondral ossification, and crucial for normal development, growth and regeneration of bone (Ivkovic et al. 2003; Kanyama et al. 2003; Lambi et al. 2012; Xu et al. 2000; Yamaai et al. 2005). Its mRNA and protein expression have been detected in normal long bones during the period of growth and (re)modeling, and have been located to osteoblasts lining metaphyseal trabeculae and those lining active, osteogenic surfaces in fracture callus (Safadi et al. 2003). CCN2 null mice show severe skeletal abnormalities involving both cartilage and bone, and die shortly after birth due to respiratory distress and cyanosis caused by severe rib cage malformations as well as by disruption of normal lung development due to reduced proliferation and increased apoptosis op pulmonary cells (Baguma-Nibasheka and Kablar 2008; Cheung et al. 2014; Falke et al. 2020; Ivkovic et al. 2003; Lambi et al. 2012; Yamaai et al. 2005).

As discussed above, increased CCN2 expression enhances the differentiation of human BM MSCs into osteoblasts (Wang et al. 2009). The osteoblast lineage-specific differentiation of MSCs is at least in part regulated by Wnt signaling and osteogenic BMPs, especially BMP-9 (Luo et al. 2004). Of note, in MSCs stimulated by Wnt3a and osteogenic BMPs, CCN2 was among the most significantly up-regulated genes (Luo et al. 2004). Prolonged CCN2 expression, in turn, inhibited both Wnt3a and BMP induced osteogenic differentiation, suggesting a regulatory role for CCN2 in normal osteogenesis (Luo et al. 2004).

The differentiation of pre-osteoblasts into bone forming osteoblasts encompasses the following phases in development: 1. proliferation, 2. maturation and extra-cellular matrix synthesis, and 3. matrix mineralization (Neve et al. 2011). A bimodal pattern of CCN2 mRNA levels is observed in primary osteoblast cultures; high CCN2 levels during the proliferative phase of early osteoblast progenitor cells (pre-osteoblasts), diminished CCN2 expression as the progenitor cells show terminal differentiation towards committed osteoblasts (Luo et al. 2004; Xu et al. 2000), and again high CCN2 mRNA levels during the mineralization phase (Safadi et al. 2003). Several studies showed that addition of rCCN2 increases the proliferation as well as mineralization of osteoblasts (Nishida et al. 2000; Safadi et al. 2003; Wang et al. 2009). Furthermore, delivery of rCCN2 into the femoral marrow cavity induced osteogenesis in vivo, which was associated with increased angiogenesis (Safadi et al. 2003; Wang et al. 2009).

Thus, CCN2 expression is important in the proliferation and differentiation of BM osteoblasts and is thereby at least indirectly of importance for the formation and maintenance of the BM microenvironment. A direct effect of CCN2 expression by osteoblasts on hematopoiesis still needs to be determined.

### CCN2 expression and function in adipocytes

Several studies indicate a regulatory role of adipocytes in hematopoiesis, although their effect might be context-dependent. Some studies indicate an inhibitory effect of BM adipocytes on hematopoiesis, as adipocyte-rich marrow spaces in mice contain less HSCs and hematopoietic progenitors cells, and adipocytes inhibit hematopoietic recovery (Ambrosi et al. 2017; Naveiras et al. 2009; Zhu et al. 2013). Another study, in contrast, shows that BM adipocytes express high levels of SCF and have a stimulatory effect on hematopoiesis, increasing hematopoietic recovery after irradiation (Zhou et al. 2017). The latter, however, seems location dependent as adipocytes in long bones promote hematopoietic recovery after irradiation, while those in the caudal vertebrae inhibit hematopoietic recovery, despite SCF production (Zhou et al. 2017). The factors responsible for these disparate observations remain to be identified.

The effect of CCN2 on adipogenesis seems inhibitory; when CCN2 is knocked down in MSCs, they differentiate into adipocytes at a six fold higher rate (Battula et al. 2013). Furthermore, when CCN2 knockdown MSCs are used to form humanized extramedullary bone, this contains less cortical bone and more adipose-like marrow tissue when compared with that derived from normal MSCs (Battula et al. 2013). The possible impact hereof on hematopoiesis still remains to be elucidated.

### CCN2 expression and function in the extracellular matrix

The ECM is a fibrillar basement network that plays a key role in cell proliferation, differentiation and migration, as well as in interactions between cells (Midwood et al. 2004). The BM ECM is composed of structural and non-structural (soluble) proteins. The structural matrix proteins include collagens, proteoglycans and other glycoproteins, the most abundant being collagens I-XI, fibronectin, laminin, tenascin, thrombospondin and elastin (Klamer and Voermans 2014). The soluble ECM proteins include growth factors, cytokines, hormones and matricellular proteins, including those of the CCN family.

CCN2 is expressed by various BM stromal cells, which include mesenchymal stem cells as well as more differentiated mesenchymal cells such as osteoblasts and CAR cells (Battula et al. 2013, 2017; Cheung et al. 2014; Igarashi et al. 2007; Istvanffy et al. 2015; Luo et al. 2004; Ren et al. 2011; Safadi et al. 2003; Schutze et al. 2005; Xu et al. 2000). Furthermore, BM stromal cells show a high level of CCN2 binding through the low density lipoprotein receptor-related protein-1 (LRP1) (Segarini et al. 2001), a well-known binding partner of CCN2. CCN2 is expected to bind many other factors in the BM as it contains many known binding partners of CCN2, such as integrins, heparan sulphate proteoglycans and growth factors. CCN2 might also induce the production of BM ECM proteins, as one study reports that BM stromal cells incubated with recombinant human CCN2 (rhCCN2) show increased expression of genes associated with ECM synthesis, including collagen type I and type III, lysyl oxidase, fibronectin, decorin and TGFβ-2 (Wells et al. 2016). It should be noted here that, typically, it has been difficult to show such activity of rCCN2 per se in other experiments.

Thus, as a matricellular protein, CCN2 can be expected to regulate intercellular signaling in the BM ECM. Furthermore, the relevance of ECM-characteristics for HSC biology and blood cell maturation underscores that CCN2 regulation might indirectly affect hematopoiesis through ECM-modification (Durand et al. 2018; Ho and Méndez-Ferrer 2020; Klamer and Voermans 2014).

## CCN2 and normal hematopoiesis

### CCN2 expression and function in hematopoietic stem cells, myelopoiesis and lymphopoiesis

CCN2 has *not* been detected in HSCs, and transplant studies showed that HSCs of *CCN2*^*−/−*^ or WT neonatal mice transplanted into WT recipients had similar HSC properties after transplantation, indicating that CCN2 indeed has minimal cell-autonomous effects in HSCs (Cheung et al. 2014). HSCs, however, *are* able to upregulate CCN2 expression in BM *stromal* cells (Istvanffy et al. 2015) and there is substantial evidence that *stromal* CCN2 does affect hematopoiesis.

The effects of stromal CCN2 on hematopoiesis have been investigated by in vitro and in vivo studies. Murine HSCs co-cultured on stroma with decreased CCN2 protein content *(shCCN2* stroma) showed reduced colony formation with increased number of hematopoietic stem and progenitor cells in G_0_ phase and senescence, and delayed time to first cell division, indicating that stromal CCN2 supports the growth and proliferation of HSCs and hematopoietic progenitor cells (Istvanffy et al. 2015). Stromal CCN2 seems to regulate the G_0_/G_1_ transition in murine HSCs by concerted action on TGF-β and WNT signaling pathways (Istvanffy et al. 2015). Furthermore, HSCs cultured on *shCCN2* stroma and subsequently transplanted into recipient WT mice in a competitive setting, showed normal initial engraftment, but at later time points (10 and 16 weeks), there was a significant decline of myeloid and B-lymphoid but not T-lymphoid engraftment (Istvanffy et al. 2015). Adding rCCN2 to these cultures partly compensated for the diminished CCN2 production by stromal cells, significantly enhancing both the number of B- and T-cells, whereas the number of myeloid cells did not change (Istvanffy et al. 2015). In addition, when the donor HSCs isolated from primary recipients were again transplanted in equal numbers into secondary recipients, none of the secondary recipients of HSCs from *shCCN2* stromal co-cultures engrafted more than 1% in the peripheral blood, BM, and spleen in contrast to control co-cultures (Istvanffy et al. 2015). Thus, co-culturing HSCs with CCN2-deficient stroma affects HSC properties in mice, with stromal CCN2 supporting HSC maintenance and longtime survival as well as supporting both myelopoiesis and B-lymphopoiesis.

CCN2 involvement in myelopoiesis and B-lymphopoiesis is also demonstrated by another study, showing that myeloid cell numbers in newborn *CCN2*^*−/−*^ mice were *decreased* in the liver, although *unaltered* in BM and spleen, whereas B-cell numbers in the same mice were *increased* in the liver, while *decreased* in BM and spleen (Cheung et al. 2014). T-cells were the same in all the compartments compared with WT mice (Cheung et al. 2014). The opposite effects of CCN2 on myelopoiesis and B-lymphopoiesis in BM and spleen versus liver might relate to differences in microenvironmental context in these compartments, although this has not been further explored. In the associated transplant study, the authors found similar results for B-cells; mice receiving liver-derived cells containing high numbers of HSCs, showed significantly *decreased* numbers of B-cells in both BM and spleen when these cells were derived from *CCN2*^*−/−*^ neonatal mice compared with that from WT mice, indicating that presence of CCN2 in the microenvironment of developing HSCs supports B-lymphopoiesis in BM and spleen (Cheung et al. 2014). As for the lower numbers of B-cells in the BM of mice receiving *CCN2*^*−/−*^ cells, pre-B and later differentiation stages were herein most affected, while pro-B populations remained unchanged and overall B-cell function was not affected (Cheung et al. 2014). This effect on B-lymphopoiesis is supported by in vitro studies, showing that CCN2, in the presence of IL-7, could potentiate B-cell proliferation and promote pro-B to pre-B cell differentiation, but not the further differentiation into sIgM + B cells (Cheung et al. 2014). In contrast to B-cells, the myeloid population in the BM of recipients transplanted with cells from *CCN2*^*−/−*^ mice was more abundant, which points to a possible inhibitory effect of CCN2 on myelopoiesis in this compartment (Cheung et al. 2014). The apparent discrepancy with the aforementioned study by Istvanffy et al., reporting that HSCs cultured on *shCCN2* stroma transplanted into recipient WT mice showed a decline of myeloid engraftment, suggesting a supportive effect of stromal CCN2 on myelopoiesis (Istvanffy et al. 2015), might relate to the differences in experimental set up.

In all, it can be concluded that stromal/environmental CCN2 is important for maintenance and longtime survival of HSCs and affects both myelopoiesis and B-lymphopoiesis. The effect on the latter two is likely to be highly dependent on the local microenvironment of the different tissue compartments and the precise effect of CCN2 on especially the myelopoiesis needs to be further determined.

### CCN2 expression in peripheral blood, megakaryopoiesis and erythropoiesis

Normal mononuclear cells derived from peripheral blood show very low to undetectable CCN2 mRNA levels (Kim et al. 1997; Sala-Torra et al. 2007; Vorwerk et al. 2000). Plasma and serum mainly contain the N-terminal fragment of CCN2, while the full-length protein is abundant in platelets and released upon platelet stimulation (Cicha et al. 2004; Kubota et al. 2004; Miyazaki et al. 2010; Roestenberg et al. 2004). The CCN2 content of a single platelet is estimated to be more than 20-fold higher than that of any other growth factor reported in platelets such as TGF-β, insulin-like growth factor-1 (IGF1), and platelet derived growth factor (PDGF)-AB (Kubota et al. 2004).

Platelets do not produce CCN2 themselves as CCN2 transcripts are absent in platelets (Gnatenko et al. 2003). Also, normal megakaryocytes do not seem to contain or produce CCN2 as neither cells from the megakaryocytic CMK cell line nor megakaryocytes differentiated from human HSCs showed any detectable CCN2 protein production or gene expression in vitro (Sumiyoshi et al. 2010). In addition, no appreciable CCN2 was detected in normal megakaryocytes in vivo (Astrom et al. 2015; Cicha et al. 2004; Sumiyoshi et al. 2010), although immunohistochemical staining indicated strong expression of CCN2 in megakaryocytes of primary myelofibrosis and in a subpopulation of megakaryocytes in patients with X-linked thrombocytopenia with thalassemia (Astrom et al. 2015). Thus, platelets are likely to take up CCN2 from the environment via endocytosis, similar to other molecules (Escolar et al. 2008), which is supported by the observation that human platelets are able to absorb exogenous CCN2 in vitro (Sumiyoshi et al. 2010). Possible receptors for endocytosis of CCN2 by platelets are LRP1 and integrin αIIbβ3, which are both known to bind CCN2 and to mediate endocytosis of their ligands (Jedsadayanmata et al. 1999; Kawata et al. 2006, 2012). The source of this platelet CCN2 is hypothesized to come from mesenchymal cells in the microenvironment, including chondrocytes and stromal fibroblasts. The transcription factor Myeloid Zinc Finger 1 (MZF1) can directly regulate CCN2 gene expression of BM stromal cells by binding to its promoter (Piszczatowski et al. 2015), and in vitro treatment of stromal fibroblasts with either vitamin A or D activates the MZF1 pathway, which increases CCN2 production and results in enhanced loading of CCN2 into developing platelets (Rozado et al. 2014). Whether other cells and factors are involved in CCN2 loading of platelets is undetermined.

To our knowledge, no data are available on the role of CCN2 in erythropoiesis.

## CCN2 and malignant hematopoiesis

CCN2 has been implicated in more than 25 different forms of cancer, mostly based on correlations (either positively or negatively) with clinical outcome (Wells et al. 2015). Altered CCN2 expression has been reported in tumor cells as well as in supporting stromal cells (Wells et al. 2015).

Several studies have investigated the role of CCN2 in leukemia. Lymphoblast from both pediatric and adult B-acute lymphoblastic leukemias (B-ALL) show moderate to highly increased CCN2 mRNA expression compared with control cells (often CD34 positive cells, CD19^+^igM^−^ cells or mononuclear cells) in the majority (60–80%) of cases (Boag et al. 2007; Sala-Torra et al. 2007; Vorwerk et al. 2000, 2002). MSCs isolated from the BM of acute myeloid leukemia (AML)-bearing mice showed increased CCN2 expression compared with MSCs from control mice (Battula et al. 2017). CCN2 expression has occasionally been described in chronic myeloid leukemia (CML) cells (Vorwerk et al. 2000), although this has not been confirmed by later studies. CCN2 gene amplification or mutations have not been reported.

### CCN2 expression, effect and regulation in acute lymphoblastic leukemias

#### CCN2 expression and prognostic effect in ALL

High CCN2 mRNA expression is frequently observed in the lymphoblasts of B-ALL, but rarely in T-ALL (Advani et al. 2010; Boag et al. 2007; Gandemer et al. 2007; Kang et al. 2010; Lu et al. 2014; Sala-Torra et al. 2007; Tesfai et al. 2012; Vorwerk et al. 2002, 2000; Welch et al. 2013, 2015). It could be hypothesized that this is related to the fact that CCN2 plays a role in normal B-cell development while no effects in T-cell development have been reported (Cheung et al. 2014).

Several studies investigated the prognostic effect of CCN2 expression in pediatric and adult B-ALL. In pediatric B-ALL, increased CCN2 expression has been associated with certain cytogenetic subgroups; B-ALL with BCR-ABL, ETV6-RUNX1 (TEL-AML1) or translocations of MLL showed high CCN2 expression (Boag et al. 2007; Gandemer et al. 2007; Tesfai et al. 2012), while those with hyperdiploidy showed low CCN2 expression, and B-ALL with an E2A-PBX1 translocation showed hardly any CCN2 expression (Boag et al. 2007). Thus, with the exception of ETV6-RUNX1, high CCN2 expression is associated with poor prognostic cytogenetics, and low/no CCN2 expression with favorable cytogenetics. In addition, high CCN2 gene expression was part of the high risk profile in a study on pediatric ALL patients with high risk features (Kang et al. 2010). This study used a 38-gene expression classifier predictive of relapse-free survival (RFS) to distinguish 2 groups, one with low relapse risk (81% 4-year RFS) and one with high relapse risk (50% 4-year RFS). Patients with *very* high-risk features (BCR-ABL1 or hypodiploidy) were excluded, as well as those with low-risk features (trisomies of chromosomes 4 or 10; t[12;21][ETV6-RUNX1]) unless they had central nervous system disease or testicular localization. In an earlier study on pediatric B-ALL, CCN2 expression of lymphoblasts at diagnosis was not found to be predictive of relapse, as the same number of patients with a relapse had CCN2 expressing lymphoblasts at diagnosis as those in continued remission (Vorwerk et al. 2002). This study, however, differed from the later published study in that it did not select for high-risk patients. Furthermore, it merely looked at absence or presence of CCN2 expression without taking the level of CCN2 expression into account. As discussed above, low CCN2 expression has been associated with favorable cytogenetics. Therefore, the level of CCN2 expression, and not merely its presence or absence, seems to be important for its prognostic value.

In adult B-ALL, higher CCN2 expression levels have also been associated with worsening of overall survival (Advani et al. 2010; Sala-Torra et al. 2007). In a study on 79 adult ALL specimens, a higher CCN2 expression level in blood or BM lymphoblasts was an independent negative predictor of survival in a multivariate proportional hazards model and correlated with the percentage of CD34 expressing blasts, although there was no correlation between CCN2 expression levels and rate of complete remission or resistant disease (Sala-Torra et al. 2007). There were also no significant differences in CCN2 expression when analyzed according to sex, age, French–American–British (FAB) classification, SWOG performance status, white blood cell counts, and number of blasts in peripheral blood or BM (Sala-Torra et al. 2007). Similar findings were reported in a smaller study on 33 adult ALL patients with relapsed or refractory disease; CCN2 expression was not predictive for complete remission rate nor for resistant disease, but there was a trend for patients with higher expression of CCN2 in circulating lymphoblasts to have an inferior overall survival (Advani et al. 2010).

Several in vitro and in vivo studies support the notion that CCN2 has a pro-leukemic effect in B-ALL. In vitro, knockdown of CCN2 in B-ALL cell lines reduces leukemia cell growth due to reduced proliferation as well as increased apoptosis (Lu et al. 2014; Wells et al. 2016). The reduced proliferation is likely due to inhibition of the G_1_/S transition, associated with decreased levels of phospho-AKT and increased levels of p27, whereas the increased apoptosis is associated with increased levels of the pro-apoptotic BCL-2 family protein BIM (Lu et al. 2014). In vivo, mice injected with genetically engineered B-ALL cells with overexpressed CCN2 showed reduced survival (Wells et al. 2016). Mice injected with B-ALL with knocked down CCN2 showed less engraftment in the BM compared with mice transplanted with control cells (B-ALL cells with empty vector) (Wells et al. 2016). Thus, reduced survival associated with elevated CCN2 expression seems related to increased engraftment, proliferation, and apoptosis resistance (Wells et al. 2016).

B-ALL cells can also secrete CCN2 (Boag et al. 2007; Welch et al. 2015; Wells et al. 2016), and addition of rhCCN2 promotes adhesion of B-ALL cells to stromal cells in vitro, which induces them to overexpress genes associated with cell cycle, intracellular transport and ECM synthesis (Wells et al. 2016). Therefore, CCN2 secreted by B-ALL cells might also enhance leukemia engraftment due to its modifying effects on the microenvironment and ECM interactions (Wells et al. 2016).

Seemingly in contrast to the above, one study showed *increased* leukemic engraftment when CCN2 was knocked down in MSCs, which suggests a protective effect of stromal CCN2, diminishing leukemic outgrowth (Battula et al. 2013). This study used CCN2 knockdown MSCs to form humanized extramedullary BM (EXM-BM) in WT mice. Increased leukemic engraftment of ALL cells was observed in this EXM-BM compared with that in the control EXM-BM derived from normal MSCs (Battula et al. 2013). This disparity suggests that not only the effects of CCN2 can be cell-type dependent, but also that the source of CCN2 might be critical (leukemic blast versus stromal cell). Another explanation might relate to the fact that actions of CCN proteins are context dependent. In the latter study, the CCN2 knockdown MSCs are used to induce newly formed bone and BM when injected together with human endothelial colony-forming cells (Battula et al. 2013). The EXM-BM derived from these CCN2 knockdown MSCs proved to be more adipocyte-rich, attributed to an inhibitory effect of CCN2 on adipogenesis, and expressed significantly higher levels of CXCL12 and of the adipocyte growth factor leptin than the EXM-BM derived from normal MSCs (Battula et al. 2013). Thus, there is not merely a down-regulation of CCN2 expression in the knockdown MSCs, but a profound change in the microenvironment they contribute to, which can be attributed to the effects of CCN2 on MSC differentiation. In particular, the enhanced leukemic engraftment in this setting might be due to the enhanced fat content of the BM with increased leptin and CXCL12 expression, possibly overruling a negative effect of CCN2 deficiency, as leptin enhances leukemic cell growth (Konopleva et al. 1999; Tabe et al. 2004) and CXCL12 is a known homing factor for leukemia cells (Möhle et al. 2000).

#### Epigenetic and post-transcriptional regulation of CCN2 in acute lymphoblastic leukemia

Both epigenetic regulation and post-transcriptional regulation of CCN2 might play a role in the pathophysiology of ALL.

DNA methylation is the only epigenetic modification of CCN2 studied in ALL. The CCN2 locus contains a dense CpG island at the 5′ end of the coding region and demethylation of this region was shown to be a common feature of pediatric B-ALL; mononuclear cells extracted from BM of these patients showed this locus to be largely unmethylated, regardless of the level of CCN2 gene expression (Welch et al. 2013). Remarkably, CD34 + cells from normal BM also showed extensive hypomethylation of the CCN2 locus, while BM lymphoblasts from T-ALL patients, not expressing detectable levels of CCN2 mRNA, showed hypermethylation focused at either end of the CCN2 CpG island (Welch et al. 2013). In B-ALL cell lines, an inverse correlation between the methylation state of the CCN2 locus and CCN2 gene expression was found: B-ALL cell lines with unmethylated CCN2 CpG islands showed high levels of CCN2 expression, while those with methylated CCN2 CpG islands showed no measurable CCN2 expression (Welch et al. 2013). In conclusion, while in B-ALL cell lines, demethylation of the CCN2 locus was associated with increased CCN2 expression, demethylation in ALL BM samples was commonly present but not related to CCN2 gene expression. Further studies are needed to elucidate the exact role of the CCN2 methylation status in ALL.

Alternative splicing of CCN2 in B-ALL is described by one study, which showed several novel short CCN2 mRNA isoforms (alternative splice forms) in B-ALL cell lines and B-ALL specimens (Welch et al. 2015). The splice forms all exhibited variable loss of sequences corresponding to exons 1–3, and in some cases loss of exon 4, but always full retention of exon 5 containing the CT domain (Welch et al. 2015). The short isoform encoding only the CT domain was the most frequently observed CCN2 alternative splice form, being present in 70% of the investigated B-ALL specimens expressing full length CCN2 (Welch et al. 2015). The shorter transcripts (but not the full length transcript) showed higher expression during the most active phase of cell growth, suggesting that they may be associated with the proliferation of B-lineage ALL cells (Welch et al. 2015). The truncated CCN2 protein with only the CT domain can still strongly bind to heparin, mediate cell adhesion and induce proliferation (Ball et al. 2003; Brigstock et al. 1997; Holbourn et al. 2009), but differences in biological activity of alternative splice products, compared to those of full length CCN2, largely remain to be elucidated.

MiRNAs play an important regulatory role in hematopoiesis and leukemogenesis (Fernandes 2017; Grobbelaar and Ford 2019; Schotte et al. 2009; Yeh et al. 2016; Yendamuri and Calin 2009; Zhang et al. 2018). The miRNA-17–92 cluster is essential for B-cell development, regulating pro-B to pre-B cell development and apoptosis of B-cells, and amplification of the miR-17–92 coding region has been associated with lymphoproliferative disease (Koralov et al. 2008; Ventura et al. 2008; Xiao et al. 2008). CCN2 is a predicted target of several miRNAs, including the miRNA-17–92 cluster (Chen et al. 2016; Ernst et al. 2010; Fox et al. 2013). It is, however, still unknown if and how miRNAs might relate CCN2 expression to ALL biology.

### CCN2 expression and effect in acute myeloid leukemia

CCN2 expression has *not* been demonstrated in AML blast cells. But similar to normal HSCs, AML cells can induce CCN2 expression in MSCs, which relies on BMP-mediated signaling (Battula et al. 2017; Li et al. 2019). The CCN2 gene in MSCs from AML-bearing mice is upregulated 12- to 33-fold across various AML genotypes compared with that in MSCs from non-AML bearing control mice (Battula et al. 2017).

The effect of stromal CCN2 on AML engraftment is not fully established. One study indicates a pro-leukemic effect; transgenic mice overexpressing CCN2 in stromal cells, injected with AML cells, show: (1) a fourfold (time-dependent) enhancement of leukemia engraftment, (2) a higher percentage of leukemia cells in the peripheral blood, and (3) more leukemia engraftment in spleens compared with WT mice (Battula et al. 2017). Another study, however, suggests an opposite effect of stromal CCN2. Increased leukemic engraftment was observed in humanized extramedullary BM (EXM-BM) in mice when this BM was formed from CCN2 knockdown MSCs (Battula et al. 2013). As has been discussed above in more detail in the section on CCN2 and ALL, the EXM-BM derived from CCN2 knockdown MSCs proved to be more adipocyte-rich, which can be attributed to the inhibitory effect of CCN2 on the adipogenic differentiation of MSCs, and expressed higher levels of leptin and CXCL12 than the EXM-BM derived from normal MSCs (Battula et al. 2013). Thus, there is not merely a down-regulation of CCN2 expression in MSCs, but a complete change in microenvironment, with more adipose tissue and higher levels of leptin and CXCL-12, factors known to enhance leukemic growth and homing, possibly overruling a negative effect of CCN2.

### CCN2 expression and effect in myeloid neoplasms with fibrosis

As described above, CCN2 enhances differentiation of cultured human BM MSCs into (myo)fibroblasts when stimulated subsequently with TGF-β (Lee et al. 2010). CCN2 is required for the differentiation of progenitor cells into contractile myofibroblasts through the regulation of extracellular matrix, cytoskeleton, cell adhesion, and cell migration genes, at least in dermal fibroblasts, as well as for the recruitment of progenitor cells to the fibrotic lesion in response to bleomycin, as has been shown by two different mouse models (Liu et al. 2014; Tsang et al. 2020; Liu et al. 2014). Another mouse fibrosis model has shown that either CCN2 mRNA or an application of exogenous CCN2 protein seems required for the development of persistent fibrosis (Mori et al. 1999). Although initially there has been some misconception that CCN2 would be merely a down-stream mediator of TGF-β, it has now been shown that CCN2 rather acts as cofactor mediating and amplifying the profibrotic actions of TGF-β through domain-specific interactions with TGF-β and its receptor (Holmes et al. 2001; Khankan et al. 2011; Mori et al. 2008), and that TGF-β and CCN2 have overlapping and distinct fibrogenic effects (Gore-Hyer et al. 2002). In a wide variety of in vivo systems, CCN2 is required for experimental fibrosis. CCN2 is important as a key central mediator of the feed-forward system that both initiates and perpetuates fibrosis since adhesive signaling/mechanotransduction, mediated by FAK and YAP/TAZ, is required for fibrosis and CCN2 activates this pathway (Dupont et al. 2011; Lachowski et al. 2018).

Higher mRNA levels of TGF-β and CCN2 have been demonstrated in BM of myelodysplastic syndrome (MDS) with fibrosis than in MDS without fibrosis (Hussein et al. 2018), suggesting a role for both in its pathophysiology. The TGF-β pathway has already been implicated in the pathogenesis of many BM disorders, including myeloid neoplasms (Bataller et al. 2019), and TGF-β is known to play a central role in the induction of BM fibrosis in myeloproliferative neoplasms (Agarwal et al. 2016). The role of CCN2 in myeloproliferative neoplasms or other BM diseases with fibrosis still needs to be elucidated.

### CCN2 expression and effect in plasma cell neoplasia

Only 1 study investigated the role of CCN2 in plasma cell neoplasia. This study reported significantly lower plasma levels of whole CCN2 in multiple myeloma (MM) patients compared with healthy controls, and in MM patients *with* bone disease compared with those *without* (Munemasa et al. 2007). Therefore, lowered CCN2 might be an indicator of bone disease in MM patients.

## Other CCN protein family members

CCN2 is just one representative of the family of closely related CCN genes, which also includes CCN1 (Cysteine rich 61/Cyr61), CCN3 (Nephroblastoma overexpressed/NOV), CCN4 (Wnt-inducible-secreted protein (WISP)-1), CCN5 (WISP-2) and CCN6 (WISP-3) (Brigstock et al. 1997; Perbal 2018).The CCN family members can work in concert to orchestrate a multitude of biological processes in similar but also partly opposite ways (Peidl et al. 2019; Perbal 2018; Riser et al. 2009, 2010). Therefore, the CCN family genes should ideally be studied together rather than separate. Unfortunately, the limited availability of well characterized tools including purified individual CCN proteins and the high complexity of their individual biologies have largely prevented such studies thus far.

As discussed in the reviews on CCN proteins in cancers and their tumor microenvironment, the role of a particular CCN protein in either potentiating or inhibiting tumor progression is related to the tumor type, and altered protein expression can be observed in either tumor cells or in tumor associated stromal cells (Wells et al. 2016; Yeger and Perbal 2016). With respect to their role in the BM microenvironment and in normal or malignant hematopoiesis, we have found no integrative studies involving multiple CCN genes. The available data on the individual non-CCN2 protein family members are discussed below and their expression in hematologic malignancies of the BM is summarized in Table 4.

**Table 4:** Expression of CCN proteins in hematologic malignancies of the bone marrow

	CCN1	CCN2	CCN3	CCN4
*B-ALL*				
Tumor cell	↑	↑	n/r	↑
Stromal cell	n/r	n/r	n/r	n/r
*T-ALL*				
Tumor cell	↑	-	n/r	n/r
Stromal cell	n/r	n/r	n/r	n/r
*CML*				
Tumor cell	↑	n/r	↓	n/r
Stromal cell	n/r	n/r	n/r	n/r
*AML*				
Tumor cell	↑	n/r	n/r	n/r
Stromal cell	↑	↑	n/r	n/r
*MM*				
Tumor cell	–/↑ ?	n/r	n/r	n/r
Stromal cell	↑	n/r	n/r	↓

Increased (↑), decreased (↓), or unaltered (–) expression of CCN proteins in tumor cells and tumor associated stromal cells as far as reported in literature

*N/R* not reported, *ALL* acute lymphoblastic leukemia, *CML* chronic myeloid leukemia, *AML* acute myeloid leukemia, *MM* multiple myeloma

### CCN1

High levels of CCN1 mRNA and protein have been demonstrated in human BM stromal cells (Djouad et al. 2007; Johnson et al. 2014; Li et al. 2012; Long et al. 2015; Schutze et al. 2005). The highest CCN1 expression is observed in MSCs, which decreases during osteogenic, adipogenic and chondrogenic differentiation (Djouad et al. 2007; Schutze et al. 2005).

**Figure 4:**
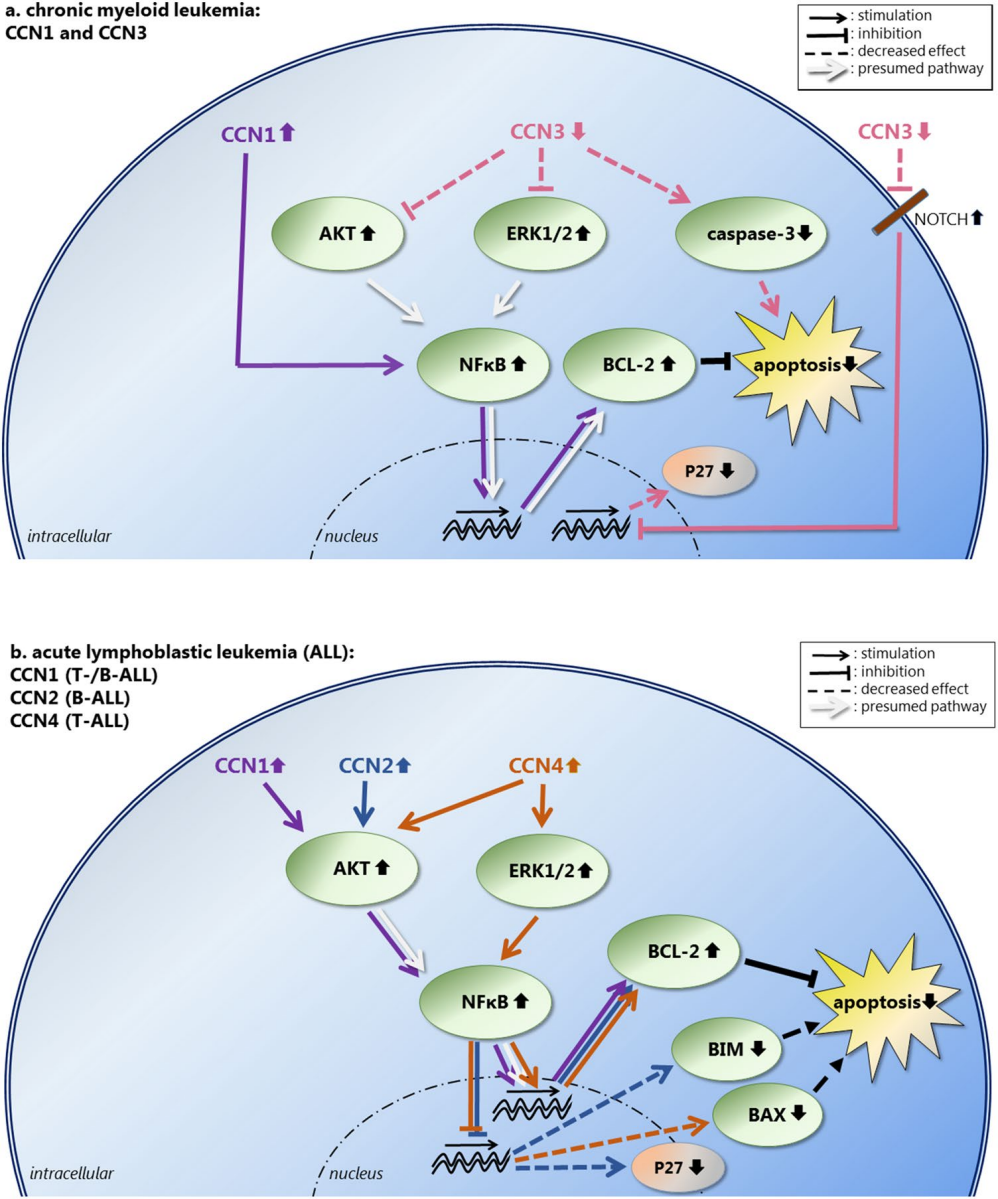
**a** Direct and indirect intracellular effects of CCN proteins in chronic myeloid leukemia (CML). CCN1 expression is increased in CML cells, inhibiting apoptosis through enhanced expression of the anti-apoptotic protein BCL2 via the NF-κB pathway, without involvement of AKT or ERK1/2. CCN3 expression is decreased in CML cells, decreasing its inhibitory effect of CCN3 on ERK and AKT phosphorylation, resulting in elevated levels of phosphorylated ERK and AKT. This leads to less apoptosis, presumably via the NF-κB pathway. In addition, decreased CCN3 levels result in less caspase 3 cleavage, thereby also reducing apoptosis. Furthermore, decreased CCN3 levels lead to less inhibition of NOTCH1 signaling, resulting in higher levels of NOTCH. This results in decreased expression of p27, disrupting cell cycle regulation. **b** Direct and indirect intracellular effects of CCN proteins in acute lymphoblastic leukemia (ALL). The effects have been demonstrated for CCN1 in B- and T-ALL, for CCN2 in B-ALL and for CCN4 in T-ALL cells. In these cells, CCN1, CCN2 as well as CCN4 expression is increased, all inhibiting apoptosis through enhanced expression of the anti-apoptotic protein BCL2. Both CCN1 and CCN2 activate AKT, whereas CCN4 activates AKT as well as ERK1/2. Involvement of the NF-κB pathway has been demonstrated for CCN1 and CCN4. In addition, increased CCN2 and CCN4 levels lead to decreased expression of the pro-apoptotic proteins BIM and BAX, respectively, both resulting in less apoptosis. Furthermore, increased CCN2 levels are associated with decreased p27 expression, thereby affecting cell cycle regulation

In (T- and B-) ALL and CML, CCN1 expression has been detected in cell lines, and increased CCN1 levels have been demonstrated in BM-derived mononuclear cells, BM aspirate supernatant, and plasma samples of ALL and CML patients (Cao et al. 2019; Song et al. 2019; Zhu et al. 2016). In ALL, BM and plasma CCN1 levels correlate with the percentage of blasts (Zhu et al. 2016). In both ALL and CML, CCN1 enhances leukemic cell survival in vitro by decreasing apoptosis through enhanced BCL2 expression via the NF-κB pathway (Song et al. 2019; Zhu et al. 2016). In ALL, AKT but not ERK1/2 affects in vitro NF-κB signaling by CCN1 (Zhu et al. 2016), whereas in CML, AKT nor ERK1/2 are involved in in vitro NF-κB signaling by CCN1 (Song et al. 2019). Direct and indirect effects of CCN1 in CML and ALL are depicted in Fig. 4a, b, respectively. CCN1 inhibition increases Imatinib-induced apoptosis of CML cells in vitro and restores the sensitivity of CML cells to Imatinib in vivo (Song et al. 2019). Similarly, CCN1 decreases Cytarabine chemosensitivity in ALL cells via NF-κB pathway activation, and inhibition of CCN1 can restore ALL cell response to Cytarabine in vitro (Cao et al. 2019). Thus, CCN1 expression of leukemic cells seems to enhance tumor cell growth as well as drug resistance in both CML and ALL.

In AML, CCN1 expression has been demonstrated in leukemic cells as well as in stromal cells (Long et al. 2015; Niu et al. 2014). CCN1 expression in AML cells was upregulated by co-culturing them with BM stromal cells (Long et al. 2015). The amount of CCN1 expression in AML cells varies, with some BM samples and cell lines showing high expression, while others having low or no detectable expression (Niu et al. 2014). CCN1 promotes growth and survival of AML cells through the MEK/ERK pathway, with up-regulation of c-Myc and Bcl-xL, an anti-apoptotic protein of the Bcl2-family, and down-regulation of Bax, a pro-apoptotic member of the Bcl-2 family (Niu et al. 2014). The β-catenin/survivin pathway does not seem to be involved (Niu et al. 2014). Inhibition of *stromal* CCN1 partially reverses the stroma-induced resistance to mitoxantrone by increasing the mitoxantrone-induced apoptosis by AML cells (Long et al. 2015), suggesting a role for CCN1 in stroma-mediated chemoresistance. Spleen tyrosine kinase (SYK) is involved in this CCN1 signaling (Long et al. 2015). Thus, in AML, CCN1 seems to have a pro-leukemic effect in both tumor and stromal cells. CCN1 gene polymophisms have been associated with either a lowered or increased risk of AML (Niu et al. 2014).

Increased CCN1 protein and gene expression have also been demonstrated in BM of patients with a plasma cell neoplasia (Johnson et al. 2014; Liu et al. 2017). Contradicting results, however, have been reported regarding cell type (plasma cells versus stromal cells) expressing CCN1 and its effect on tumor cell growth. Johnson et al. found high CCN1 gene expression in multiple myeloma (MM) associated BM *stromal* cells, but no CCN1 expression in cultured (normal or malignant) plasma cells (Johnson et al. 2014), whereas Dotterweich et al. *did* demonstrated CCN1 mRNA and protein in the cells of their MM cell line, which could be markedly increased by MSC contact or addition of recombinant CCN1 (Dotterweich et al. 2014). And whereas Johnson et al. showed recombinant CCN1 to inhibit growth of MM cells (Johnson et al. 2014), the viability of primary myeloma cells in the study of Dotterweich et al. increased significantly after CCN1 incubation, implying a pro-myeloma effect of CCN1 (Dotterweich et al. 2014). The contradicting results of CCN1 on MM cells might be explained by the use of different cell lines (H929 versus INA-6 MM cell line), difference between cell lines versus patient samples, and differences in experimental conditions as the effects of CCN proteins are known to be dependent on the micro-environment including the presence of certain cytokines.

Two studies imply that CCN1 has a favorable clinical effect in plasma cell neoplasms; Johnson et al. showed elevated serum CCN1 levels to be associated with a longer time to progression of monoclonal gammopathy of undetermined significance (MGUS) to overt MM and as such with a superior overall survival (Johnson et al. 2014), and Liu et al. showed increased CCN1 protein levels in the BM to be inversely associated with the severity of myeloma associated bone lesions (Liu et al. 2017). In addition, both studies confirmed in mouse models that overexpressed CCN1 in engrafted MM cells results in reduced bone disease (Johnson et al. 2014; Liu et al. 2017). This effect seems opposite to that of CCN2, showing decreased levels in MM patients with bone disease (Munemasa et al. 2007).

### CCN3

CCN3 gene expression has been demonstrated in HSCs and hematopoietic progenitor cells (Bruno et al. 2004; Gupta et al. 2007; Ishihara et al. 2014; Kimura et al. 2010) as well as in MSCs (Djouad et al. 2007). In MSCs, CCN3 expression increases two- to threefold after chondrogenesis (Djouad et al. 2007). In HSCs, CCN3 is essential for self-renewal, which seems to be at least in part due to its effect on cell cycling and NOTCH signaling (Gupta et al. 2007).

Similar to the other CCN family members, expression of CCN3 is strongly influenced by cytokines from the microenvironment. Without stimulation, HSCs show low levels of CCN3, but its expression can increase over 100-fold upon stimulation (Kimura et al. 2010). Interleukin 3 (IL-3) is the key cytokine for CCN3 induction in HSCs, directly increasing CCN3 expression by inducing STAT5A/B binding to a γ-interferon-activated sequences site in the CCN3 gene promoter (Kimura et al. 2010). Furthermore, exogenous CCN3 can induce endogenous expression of CCN3 in HSCs by binding to integrin αvβ3 on their cell surface, thereby affecting the long-term repopulating activity of HSCs (Ishihara et al. 2014). This process is context dependent; TPO mediates CCN3 binding to integrin αvβ3, inducing CCN3 expression likely through STAT5 activation, thereby supporting the long-term repopulating activity of HSCs (Ishihara et al. 2014). IFN-γ, however, impairs this TPO-induced expression of CCN3, likely through the activation of STAT1, inhibiting the long-term repopulating activity of HSCs (Ishihara et al. 2014).

In the transplant setting, exposure of umbilical cord blood to CCN3 can enhance engraftment by increased recruitment of cells with the highest long-term HSCs function, as these are preferentially bound to CCN3 through integrin α6 (a.k.a. CD49f) (Gupta et al. 2007).

In CML, the CCN3 gene is down-regulated and its protein expression decreased as a direct consequence of the BCR-ABL tyrosine kinase activity, mediated at least in part by microRNAs 130a/b (McCallum et al. 2006; Suresh et al. 2011). The decreased cellular CCN3 protein content was shown to correlate with increased CCN3 secretion (McCallum et al. 2006). Treatment with Imatinib increases CCN3 expression (McCallum et al. 2006). On the other hand, overexpression of CCN3 by transfection or treatment with the recombinant protein, significantly reduces tumor cell growth by enhancing Imatinib induced apoptosis (McCallum et al. 2009, 2012). CCN3 induced reduced cell growth and enhanced apoptosis is associated with enhanced caspase 3 cleavage and reduced phosphorylation of ERK and AKT (McCallum et al. 2009, 2012). Furthermore, CCN3 upregulates the expression of β4 integrin, which might affect the re-installation of growth control mechanisms (McCallum et al. 2012). In addition, CCN3 inhibits NOTCH1 signaling in CML, which is associated with increased expression of p27, thereby contributing to the restoration of cell cycle regulation (Suresh et al. 2013). Thus, the oncogenic BCR-ABL protein in CML enhances cell survival at least in part through its inhibitory effect on CCN3, resulting in reduced apoptosis and enhanced cell growth. These direct and indirect effects of CCN3 in CML are depicted in Fig. 4a.

A possible pathogenic role for the increased amount of secreted CCN3 needs to be determined. Furthermore, methylation of the CCN3 promoter was significantly increased in peripheral blood samples of CML patients compared with healthy controls, but this was unaffected by Imatinib treatment (Vatanmakanian et al. 2019). Whether hypermethylation of CCN3 is important in the pathophysiology in CML needs to be further investigated.

### CCN4–6

CCN4, CCN5 and CCN6 expression has been demonstrated in MSCs (Djouad et al. 2007; Schutze et al. 2005). Like CCN3, CCN4 expression increases after chondrogenesis, whereas CCN6 expression decreases (Djouad et al. 2007; Schutze et al. 2005). CCN5 expression declines during adipogenic differentiation (Schutze et al. 2005).

Two studies investigated CCN4 in malignant hematopoiesis. The first describes a threefold decrease in mRNA expression of CCN4 in BM MSCs in MM compared with healthy age-matched controls, which meaning remains to be established (Corre et al. 2007). The other study shows variable and sometimes high mRNA and protein expression of CCN4 in T-ALL cell lines (Zhang et al. 2015). Knockdown of CCN4 in the cell line with the highest expression inhibited proliferation and induced apoptosis by down-regulating expression of, amongst others, p-AKT, p-ERK and Bcl-2, and upregulation of Bax, suggesting a possible pro-leukemic effect of CCN4 in T-ALL (Fig. 4b) (Zhang et al. 2015). We found no studies on CCN5 or CCN6 with regard to normal or malignant hematopoiesis.

### Summary of CCN protein family members

In summary, the collected data on CCN1-6 with respect to BM microenvironment and normal or malignant hematopoiesis show, as far as the different experimental approaches allow such a comparison, many similarities and some differences between the CCN family members. In normal BM, expression of all CCN proteins have been demonstrated in MSCs, often at high levels and declining during lineage commitment and further differentiation, whereas only CCN3 expression has been found in HSCs. CCN3 directly affects the maintenance of HSCs, while CCN2 indirectly supports it through its secretion by MSCs. In hematologic malignancies of the BM, the CCN family members are often altered in either tumor cells or in the stromal cells (Table 4), usually, but not always, displaying pro-tumor effects. Many aspects of the CCN proteins in the various hematologic malignancies, however, remain to be elucidated.

## CCN2 therapeutic options

Targeting CCN2 may be a therapeutic option for diseases associated with increased CCN2 expression, malignant as well as non-malignant. CCN2 can be inhibited by:

1. The use of an anti-CCN2 antibody (Aikawa et al. 2006; Alapati et al. 2011; Barbe et al. 2020a, b; Bickelhaupt et al. 2017; Dornhofer et al. 2006; Finger et al. 2014; Makino et al. 2017; Moran-Jones et al. 2015; Neesse et al. 2013; Ohara et al. 2018; Raghu et al. 2016; Richeldi et al. 2020; Sakai et al. 2017),
2. Gene expression silencing by antisense oligonucleotides (ASOs) or small interfering RNAs (siRNAs) (Chen et al. 2014; Gale et al. 2018; Gibson et al. 2017; Jensen et al. 2018; Kang et al. 2020; Li et al. 2006; Okada et al. 2005; Sisco et al. 2008; Sung et al. 2013; Yokoi et al. 2004; Yoon et al. 2016),
3. Drugs that indirectly (and less specifically) inhibit CCN2 expression, for example by targeting Sirtuin 1 (Sirt1) (Ren et al. 2017), peroxisome proliferator-activated receptor gamma (PPARγ) (Sun et al. 2006; Zhao et al. 2006), the CCN2 transcriptional regulators YAP and TAZ (Ji et al. 2018), or proteins involved in signaling pathways affecting CCN2 transcription such FAK (Peidl et al. 2019),
4. The use of CCN3, as it can antagonize the effects of CCN2 (Peidl et al. 2019; Riser et al. 2009, 2010, 2014).

Of the above, only the use of an anti-CCN2 antibody and the use of ASOs and siRNAs have made it to clinical trials for their direct effect on CCN2 (Gale et al. 2018; Jensen et al. 2018; https://www.prnewswire.com/news-releases/rxi-pharmaceuticals-announces-positive-results-from-phase-12-trial-with-rxi-109-for-retinal-scarring-300690078.html; http://www.sciencedirect.com/science/article/pii/S0190962214008147). The PPARγ agonist Rosiglitazone is an FDA approved drug for the treatment of type 2 diabetes mellitus and has anti-fibrotic effects with in vitro and in vivo reduction of CCN2 expression in different organs (Gao et al. 2007; Guo et al. 2012; Ihm et al. 2010; Jeon et al. 2015). The FAK inhibitor GSK2256098 is and has been tested in phase 1 and 2 studies for pulmonary hypertension and solid tumors, respectively (http://www.clinicaltrials.gov), whereas the FAK inhibitor PF573228 blocks CCN2 expression in vitro (Peidl et al. 2019). Several Sirt1 activators have been tested in clinical trials as reviewed by Dai et al. (2018), although their suppressive effects on CCN2 expression have only been demonstrated in animal models (Ren et al. 2017). The Hippo pathway is important in oncogenesis and many currently used drugs restrict YAP/TAZ activities, whereas several novel YAP/TAZ inhibitors are under development, as is reviewed by Pobbati and Hong (2020). The use of CCN3 as a counter-regulator and a potential therapeutic agent has thus far been tested in experimental models (Riser et al. 2014).

The only therapeutic option for targeting CCN2 that has been studied in BM diseases, is the use of the humanized monoclonal anti-CCN2 antibody FG-3019 (Pamrevlumab), which, in combination with conventional chemotherapy, significantly prolonged the survival of mice injected with a primary xenograft of B-ALL cells (Lu et al. 2014). Pamrevlumab showed a very favorable clinical safety profile in a phase 2 study of idiopathic pulmonary fibrosis (Raghu et al. 2016; Richeldi et al. 2020). Two other phase 2 studies, on Pamrevlumab in Duchenne muscular dystrophy [https://clinicaltrials.gov/ct2/show/NCT02606136] and on Pamrevlumab in hospitalized patients with acute COVID-19 disease [https://clinicaltrials.gov/show/NCT04432298], are ongoing. Furthermore, three phase 3 studies, including one on locally advanced pancreatic cancer, have started [https://clinicaltrials.gov/ct2/show/NCT03955146; https://clinicaltrials.gov/ct2/show/NCT04371666 and https://clinicaltrials.gov/ct2/show/NCT03941093], while a fourth one is announced [https://clinicaltrials.gov/ct2/show/NCT04419558]. Pamrevlumab has neither been tested in clinical trials on ALL, nor in other BM diseases.

## Concluding remarks

The BM microenvironment is a complex and not fully unraveled milieu made up of many different cell populations, structural matrix proteins and soluble factors communicating with each other and forming different niches essential for normal hematopoiesis. It is subject to modulation by many different factors. One of these is CCN2, a matricellular protein with a wide variety of functions, known to be important in ECM for both the production of ECM proteins and the coordination of signaling pathways. CCN2 is produced by MSCs and other mesenchymal cells of the BM. It is involved in many different aspects of MSC biology, including proliferation, migration and differentiation. In addition, it plays a role in normal B-cell development and has been implicated in the maintenance and longtime survival of HSCs. Furthermore, CCN2 is shown to be overexpressed in leukemic cells of B-ALL, the most studied BM disease in this regard, in which it is associated with reduced overall survival. In AML samples, increased CCN2 expression is demonstrated in MSCs, which also affects leukemic engraftment, but in a way that still needs to be determined.

All other CCN family members are expressed in MSCs as well, whereas only CCN3 expression has been demonstrated in HSCs. Except for CCN5 and CCN6, which have not been studied in this context, all CCN protein family members have been associated with hematologic malignancies and often, but not always, their expression is increased in either tumor cells or in stromal cells, mostly displaying pro-tumor effects.

With this review, the authors hope to increase awareness of the CCN proteins, especially CCN2, as important players in the BM and as attractive subject for further studies on the BM microenvironment and BM diseases. These studies should include the use of anti-CCN therapies for their effect in neoplastic diseases such as ALL, but also for their possible disease modifying activities, for example on BM fibrosis.

## References

[CR1] Abd El Kader T, Kubota S, Nishida T, Hattori T, Aoyama E, Janune D, Hara ES, Ono M, Tabata Y, Kuboki T, Takigawa M (2014). The regenerative effects of CCN2 independent modules on chondrocytes in vitro and osteoarthritis models in vivo. Bone.

[CR2] Abou-Shady M, Friess H, Zimmermann A, Di Mola FF, Guo XZ, Baer HU, Büchler MW (2000). Connective tissue growth factor in human liver cirrhosis. Liver.

[CR3] Abraham DJ, Shiwen X, Black CM, Sa S, Xu Y, Leask A (2000). Tumor necrosis factor alpha suppresses the induction of connective tissue growth factor by transforming growth factor-beta in normal and scleroderma fibroblasts. J Biol Chem.

[CR4] Abreu JG, Ketpura NI, Reversade B, De Robertis EM (2002). Connective-tissue growth factor (CTGF) modulates cell signalling by BMP and TGF-beta. Nat Cell Biol.

[CR5] Acar M, Kocherlakota KS, Murphy MM, Peyer JG, Oguro H, Inra CN, Jaiyeola C, Zhao Z, Luby-Phelps K, Morrison SJ (2015). Deep imaging of bone marrow shows non-dividing stem cells are mainly perisinusoidal. Nature.

[CR6] Advani AS, Gundacker HM, Sala-Torra O, Radich JP, Lai R, Lovak ML, Lancet JE, Coutre SE, Stuart RK, Mims MP, Stiff PJ, Appelbaum FR (2010). Southwest Oncology Group Study S0530: a phase 2 trial of clofarabine and cytarabine for relapsed or refractory acute lymphocytic leukaemia. Br J Haematol.

[CR7] Agarwal A, Morrone K, Bartenstein M, Zhao ZJ, Verma A, Goel S (2016). Bone marrow fibrosis in primary myelofibrosis: pathogenic mechanisms and the role of TGF-beta. Stem Cell Investig.

[CR8] Aikawa T, Gunn J, Spong SM, Klaus SJ, Korc M (2006). Connective tissue growth factor-specific antibody attenuates tumor growth, metastasis, and angiogenesis in an orthotopic mouse model of pancreatic cancer. Mol Cancer Ther.

[CR9] Akashi S, Nishida T, El-Seoudi A, Takigawa M, Iida S, Kubota S (2018). Metabolic regulation of the CCN family genes by glycolysis in chondrocytes. J Cell Commun Signal.

[CR10] Alapati D, Rong M, Chen S, Hehre D, Rodriguez MM, Lipson KE, Wu S (2011). Connective tissue growth factor antibody therapy attenuates hyperoxia-induced lung injury in neonatal rats. Am J Respir Cell Mol Biol.

[CR11] Ambrosi TH, Scialdone A, Graja A, Gohlke S, Jank AM, Bocian C, Woelk L, Fan H, Logan DW, Schurmann A, Saraiva LR, Schulz TJ (2017). Adipocyte accumulation in the bone marrow during obesity and aging impairs stem cell-based hematopoietic and bone regeneration. Cell Stem Cell.

[CR12] Anthony BA, Link DC (2014). Regulation of hematopoietic stem cells by bone marrow stromal cells. Trends Immunol.

[CR13] Aoyama E, Hattori T, Hoshijima M, Araki D, Nishida T, Kubota S, Takigawa M (2009). N-terminal domains of CCN family 2/connective tissue growth factor bind to aggrecan. Biochem J.

[CR14] Aoyama E, Kubota S, Takigawa M (2012). CCN2/CTGF binds to fibroblast growth factor receptor 2 and modulates its signaling. FEBS Lett.

[CR15] Aoyama E, Kubota S, Khattab HM, Nishida T, Takigawa M (2015). CCN2 enhances RANKL-induced osteoclast differentiation via direct binding to RANK and OPG. Bone.

[CR16] Arranz L, Sanchez-Aguilera A, Martin-Perez D, Isern J, Langa X, Tzankov A, Lundberg P, Muntion S, Tzeng YS, Lai DM, Schwaller J, Skoda RC, Mendez-Ferrer S (2014). Neuropathy of haematopoietic stem cell niche is essential for myeloproliferative neoplasms. Nature.

[CR17] Asada N, Kunisaki Y, Pierce H, Wang Z, Fernandez NF, Birbrair A, Ma'ayan A, Frenette PS (2017). Differential cytokine contributions of perivascular haematopoietic stem cell niches. Nat Cell Biol.

[CR18] Astrom M, Hahn-Stromberg V, Zetterberg E, Vedin I, Merup M, Palmblad J (2015). X-linked thrombocytopenia with thalassemia displays bone marrow reticulin fibrosis and enhanced angiogenesis: comparisons with primary myelofibrosis. Am J Hematol.

[CR19] Aurrand-Lions M, Mancini SJC (2018). Murine bone marrow niches from hematopoietic stem cells to B cells. Int J Mol Sci.

[CR20] Babic AM, Chen C-C, Lau LF (1999). Fisp12/mouse connective tissue growth factor mediates endothelial cell adhesion and migration through integrin alphavbeta3, promotes endothelial cell survival, and induces angiogenesis in vivo. Molec Cell Biol.

[CR21] Baguma-Nibasheka M, Kablar B (2008). Pulmonary hypoplasia in the connective tissue growth factor (Ctgf) null mouse. Dev Dyn.

[CR22] Bai KJ, Chen BC, Pai HC, Weng CM, Yu CC, Hsu MJ, Yu MC, Ma HP, Wu CH, Hong CY, Kuo ML, Lin CH (2013). Thrombin-induced CCN2 expression in human lung fibroblasts requires the c-Src/JAK2/STAT3 pathway. J Leukoc Biol.

[CR23] Balderman SR, Li AJ, Hoffman CM, Frisch BJ, Goodman AN, LaMere MW, Georger MA, Evans AG, Liesveld JL, Becker MW, Calvi LM (2016). Targeting of the bone marrow microenvironment improves outcome in a murine model of myelodysplastic syndrome. Blood.

[CR24] Ball DK, Rachfal AW, Kemper SA, Brigstock DR (2003). The heparin-binding 10 kDa fragment of connective tissue growth factor (CTGF) containing module 4 alone stimulates cell adhesion. J Endocrinol.

[CR25] Barbe MF, Hilliard BA, Amin M, Harris MY, Hobson LJ, Cruz GE, Dorotan JT, Paul RW, Klyne DM, Popoff SN (2020). Blocking CTGF/CCN2 reverses neural fibrosis and sensorimotor declines in a rat model of overuse-induced median mononeuropathy. J Orthop Res.

[CR26] Barbe MF, Hilliard BA, Amin M, Harris MY, Hobson LJ, Cruz GE, Popoff SN (2020). Blocking CTGF/CCN2 reduces established skeletal muscle fibrosis in a rat model of overuse injury. FASEB J.

[CR27] Bataller A, Montalban-Bravo G, Soltysiak KA, Garcia-Manero G (2019). The role of TGFbeta in hematopoiesis and myeloid disorders. Leukemia.

[CR28] Battula VL, Chen Y, Cabreira Mda G, Ruvolo V, Wang Z, Ma W, Konoplev S, Shpall E, Lyons K, Strunk D, Bueso-Ramos C, Davis RE, Konopleva M, Andreeff M (2013). Connective tissue growth factor regulates adipocyte differentiation of mesenchymal stromal cells and facilitates leukemia bone marrow engraftment. Blood.

[CR29] Battula VL, Le PM, Sun JC, Nguyen K, Yuan B, Zhou X, Sonnylal S, McQueen T, Ruvolo V, Michel KA, Ling X, Jacamo R, Shpall E, Wang Z, Rao A, Al-Atrash G, Konopleva M, Davis RE, Harrington MA, Cahill CW, Bueso-Ramos C, Andreeff M (2017). AML-induced osteogenic differentiation in mesenchymal stromal cells supports leukemia growth. JCI Insight.

[CR30] Behnes M, Brueckmann M, Lang S, Weiss C, Ahmad-Nejad P, Neumaier M, Borggrefe M, Hoffmann U (2014). Connective tissue growth factor (CTGF/CCN2): diagnostic and prognostic value in acute heart failure. Clin Res Cardiol.

[CR31] Bickelhaupt S, Erbel C, Timke C, Wirkner U, Dadrich M, Flechsig P, Tietz A, Pfohler J, Gross W, Peschke P, Hoeltgen L, Katus HA, Grone HJ, Nicolay NH, Saffrich R, Debus J, Sternlicht MD, Seeley TW, Lipson KE, Huber PE (2017). Effects of CTGF blockade on attenuation and reversal of radiation-induced pulmonary fibrosis. J Natl Cancer Inst.

[CR32] Blalock TD, Gibson DJ, Duncan MR, Tuli SS, Grotendorst GR, Schultz GS (2012). A connective tissue growth factor signaling receptor in corneal fibroblasts. Invest Ophthalmol Vis Sci.

[CR33] Blau O, Hofmann WK, Baldus CD, Thiel G, Serbent V, Schumann E, Thiel E, Blau IW (2007). Chromosomal aberrations in bone marrow mesenchymal stroma cells from patients with myelodysplastic syndrome and acute myeloblastic leukemia. Exp Hematol.

[CR34] Boag JM, Beesley AH, Firth MJ, Freitas JR, Ford J, Brigstock DR, de Klerk NH, Kees UR (2007). High expression of connective tissue growth factor in pre-B acute lymphoblastic leukaemia. Br J Haematol.

[CR35] Bork P (1993). The modular architecture of a new family of growth regulators related to connective tissue growth factor. FEBS Lett.

[CR36] Bornstein P (1995). Diversity of function is inherent in matricellular proteins: an appraisal of thrombospondin I. J Cell Biol.

[CR37] Bornstein P (2009). Matricellular proteins: an overview. J Cell Commun Signal.

[CR38] Bornstein P, Sage EH (2002). Matricellular proteins: extracellular modulators of cell function. Curr Opin Cell Biol.

[CR39] Boyerinas B, Zafrir M, Yesilkanal AE, Price TT, Hyjek EM, Sipkins DA (2013). Adhesion to osteopontin in the bone marrow niche regulates lymphoblastic leukemia cell dormancy. Blood.

[CR40] Bradham DM, Igarashi A, Potter RL, Grotendorst GR (1991). Connective tissue growth factor: a cysteine-rich mitogen secreted by human vascular endothelial cells is related to the SRC-induced immediate early gene product CEF-10. J Cell Biol.

[CR41] Brigstock DR (1999). The connective tissue growth factor/cysteine-rich 61/nephroblastoma overexpressed (CCN) family. Endocr Rev.

[CR42] Brigstock DR, Steffen CL, Kim GY, Vegunta RK, Diehl JR, Harding PA (1997). Purification and characterization of novel heparin-binding growth factors in uterine secretory fluids. J Biol Chem.

[CR43] Brigstock DR, Goldschmeding R, Katsube K-i, Lam SCT, Lau LF, Lyons K, Naus C, Perbal B, Riser B, Takigawa M, Yeger H (2003). Proposal for a unified CCN nomenclature. J Clin Pathol Mol Pathol.

[CR44] Brunner A, Chinn J, Neubauer M, Purchio AF (1991). Identification of a gene family regulated by transforming growth factor-beta DNA. Cell Biol.

[CR45] Bruno L, Hoffmann R, McBlane F, Brown J, Gupta R, Joshi C, Pearson S, Seidl T, Heyworth C, Enver T (2004). Molecular signatures of self-renewal, differentiation, and lineage choice in multipotential hemopoietic progenitor cells in vitro. Mol Cell Biol.

[CR46] Cai Y, Huang G, Ma L, Dong L, Chen S, Shen X, Zhang S, Xue R, Sun D, Zhang S (2018). Smurf2, an E3 ubiquitin ligase, interacts with PDE4B and attenuates liver fibrosis through miR-132 mediated CTGF inhibition. Biochim Biophys Acta Mol Cell Res.

[CR47] Cao Y, Wu C, Song Y, Lin Z, Kang Y, Lu P, Zhang C, Huang Q, Hao T, Zhu X, Hu J (2019). Cyr61 decreases Cytarabine chemosensitivity in acute lymphoblastic leukemia cells via NF-kappaB pathway activation. Int J Mol Med.

[CR48] Chambers RC, Leoni P, Blanc-Brude OP, Wembridge DE, Laurent GJ (2000). Thrombin is a potent inducer of connective tissue growth factor production via proteolytic activation of protease-activated receptor-1. J Biol Chem.

[CR49] Chaqour B (2020). Caught between a "Rho" and a hard place: are CCN1/CYR61 and CCN2/CTGF the arbiters of microvascular stiffness?. J Cell Commun Signal.

[CR50] Charrier A, Chen R, Chen L, Kemper S, Hattori T, Takigawa M, Brigstock DR (2014). Connective tissue growth factor (CCN2) and microRNA-21 are components of a positive feedback loop in pancreatic stellate cells (PSC) during chronic pancreatitis and are exported in PSC-derived exosomes. J Cell Commun Signal.

[CR51] Charrier A, Chen R, Kemper S, Brigstock DR (2014). Regulation of pancreatic inflammation by connective tissue growth factor (CTGF/CCN2). Immunology.

[CR52] Chasis JA, Mohandas N (2008). Erythroblastic islands: niches for erythropoiesis. Blood.

[CR53] Che H, Wang Y, Li Y, Lv J, Li H, Liu Y, Dong R, Sun Y, Xu X, Zhao J, Wang L (2019). Inhibition of microRNA-150–5p alleviates cardiac inflammation and fibrosis via targeting Smad7 in high glucose-treated cardiac fibroblasts. J Cell Physiol.

[CR54] Chen CC, Lau LF (2009). Functions and mechanisms of action of CCN matricellular proteins. Int J Biochem Cell Biol.

[CR55] Chen CC, Chen N, Lau LF (2001). The angiogenic factors Cyr61 and connective tissue growth factor induce adhesive signaling in primary human skin fibroblasts. J Biol Chem.

[CR56] Chen L, Charrier A, Zhou Y, Chen R, Yu B, Agarwal K, Tsukamoto H, Lee LJ, Paulaitis ME, Brigstock DR (2014). Epigenetic regulation of connective tissue growth factor by MicroRNA-214 delivery in exosomes from mouse or human hepatic stellate cells. Hepatology.

[CR57] Chen YC, Chen BC, Yu CC, Lin SH, Lin CH (2016). miR-19a, -19b, and -26b Mediate CTGF expression and pulmonary fibroblast differentiation. J Cell Physiol.

[CR58] Chen JQ, Ou YL, Huang ZP, Hong YG, Tao YP, Wang ZG, Ni JS, Hao LQ, Lin H (2019). MicroRNA-212–3p inhibits the Proliferation and Invasion of Human Hepatocellular Carcinoma Cells by Suppressing CTGF expression. Sci Rep.

[CR59] Cheung LC, Strickland DH, Howlett M, Ford J, Charles AK, Lyons KM, Brigstock DR, Goldschmeding R, Cole CH, Alexander WS, Kees UR (2014). Connective tissue growth factor is expressed in bone marrow stromal cells and promotes interleukin-7-dependent B lymphopoiesis. Haematologica.

[CR60] Chowdhury I, Chaqour B (2004). Regulation of connective tissue growth factor (CTGF/CCN2) gene transcription and mRNA stability in smooth muscle cells. Involvement of RhoA GTPase and p38 MAP kinase and sensitivity to actin dynamics. Eur J Biochem.

[CR61] Cicha I, Goppelt-Struebe M (2009). Connective tissue growth factor: context-dependent functions and mechanisms of regulation. BioFactors.

[CR62] Cicha I, Garlichs CD, Daniel WG, Goppelt-Struebe M (2004). Activated human platelets release connective tissue growth factor. Thromb Haemost.

[CR63] Cooker LA, Peterson D, Rambow J, Riser ML, Riser RE, Najmabadi F, Brigstock D, Riser BL (2007). TNF-alpha, but not IFN-gamma, regulates CCN2 (CTGF), collagen type I, and proliferation in mesangial cells: possible roles in the progression of renal fibrosis. Am J Physiol Renal Physiol.

[CR64] Corre J, Mahtouk K, Attal M, Gadelorge M, Huynh A, Fleury-Cappelleso S, Danho C, Laharrague P, Klein B, Rème T, Bourin P (2007). Bone marrow mesenchymal stem cells are abnormal in multiple myeloma. Leukemia.

[CR65] Dai H, Sinclair DA, Ellis JL, Steegborn C (2018). Sirtuin activators and inhibitors: promises, achievements, and challenges. Pharmacol Ther.

[CR66] Dammeier J, Beer HD, Brauchle M, Werner S (1998). Dexamethasone is a novel potent inducer of connective tissue growth factor expression. implications for glucocorticoid therapy. J Biol Chem.

[CR67] de Winter P, Leoni P, Abraham D (2008). Connective tissue growth factor: structure-function relationships of a mosaic, multifunctional protein. Growth Factors.

[CR68] Dean RA, Butler GS, Hamma-Kourbali Y, Delbe J, Brigstock DR, Courty J, Overall CM (2007). Identification of candidate angiogenic inhibitors processed by matrix metalloproteinase 2 (MMP-2) in cell-based proteomic screens: disruption of vascular endothelial growth factor (VEGF)/heparin affin regulatory peptide (pleiotrophin) and VEGF/Connective tissue growth factor angiogenic inhibitory complexes by MMP-2 proteolysis. Mol Cell Biol.

[CR69] di Mola FF, Friess H, Martignoni ME, Di Sebastiano P, Zimmermann A, Innocenti P, Graber H, Gold LI, Korc M, Büchler MW (1999). Connective tissue growth factor is a regulator for fibrosis in human chronic pancreatitis. Ann Surg.

[CR70] di Mola FF, Di Sebastiano P, Gardini A, Innocenti P, Zimmermann A, Buchler MW, Friess H (2004). Differential expression of connective tissue growth factor in inflammatory bowel disease. Digestion.

[CR71] Ding L, Morrison SJ (2013). Haematopoietic stem cells and early lymphoid progenitors occupy distinct bone marrow niches. Nature.

[CR72] Djouad F, Delorme B, Maurice M, Bony C, Apparailly F, Louis-Plence P, Canovas F, Charbord P, Noel D, Jorgensen C (2007). Microenvironmental changes during differentiation of mesenchymal stem cells towards chondrocytes. Arthritis Res Ther.

[CR73] Dornhofer N, Spong S, Bennewith K, Salim A, Klaus S, Kambham N, Wong C, Kaper F, Sutphin P, Nacamuli R, Hockel M, Le Q, Longaker M, Yang G, Koong A, Giaccia A (2006). Connective tissue growth factor-specific monoclonal antibody therapy inhibits pancreatic tumor growth and metastasis. Cancer Res.

[CR74] Dotterweich J, Ebert R, Kraus S, Tower RJ, Jakob F, Schutze N (2014). Mesenchymal stem cell contact promotes CCN1 splicing and transcription in myeloma cells. Cell Commun Signal.

[CR75] Duncan MR, Frazier KS, Abramson S, Williams S, Klapper H, Huang X, Grotendorst GR (1999). Connective tissue growth factor mediates transforming growth factor beta-induced collagen synthesis: down-regulation by cAMP. FASEB J.

[CR76] Dupont S, Morsut L, Aragona M, Enzo E, Giulitti S, Cordenonsi M, Zanconato F, Le Digabel J, Forcato M, Bicciato S, Elvassore N, Piccolo S (2011). Role of YAP/TAZ in mechanotransduction. Nature.

[CR77] Durand C, Charbord P, Jaffredo T (2018). The crosstalk between hematopoietic stem cells and their niches. Curr Opin Hematol.

[CR78] Edwards LA, Woolard K, Son MJ, Li A, Lee J, Ene C, Mantey SA, Maric D, Song H, Belova G, Jensen RT, Zhang W, Fine HA (2011). Effect of brain- and tumor-derived connective tissue growth factor on glioma invasion. J Natl Cancer Inst.

[CR79] Eltoukhy HS, Sinha G, Moore C, Guiro K, Rameshwar P (2016). CXCL12-abundant eeticular (CAR) cells: a review of the literature with relevance to cancer stem cell survival. J Cancer Stem Cell Res.

[CR80] Ernst A, Campos B, Meier J, Devens F, Liesenberg F, Wolter M, Reifenberger G, Herold-Mende C, Lichter P, Radlwimmer B (2010). De-repression of CTGF via the miR-17–92 cluster upon differentiation of human glioblastoma spheroid cultures. Oncogene.

[CR81] Escolar G, Lopez-Vilchez I, Diaz-Ricart M, White JG, Galan AM (2008). Internalization of tissue factor by platelets. Thromb Res.

[CR82] Faherty N, Curran SP, O'Donovan H, Martin F, Godson C, Brazil DP, Crean JK (2012). CCN2/CTGF increases expression of miR-302 microRNAs, which target the TGFbeta type II receptor with implications for nephropathic cell phenotypes. J Cell Sci.

[CR83] Falke LL, He N, de Sousa C, Lopes SM, Broekhuizen R, Lyons K, Nguyen TQ, Goldschmeding R (2020). FoxD1-driven CCN2 deletion causes axial skeletal deformities, pulmonary hypoplasia, and neonatal asphyctic death. J Cell Commun Signal.

[CR84] Fan WH, Karnovsky MJ (2002). Increased MMP-2 expression in connective tissue growth factor over-expression vascular smooth muscle cells. J Biol Chem.

[CR85] Fernandes Q (2017). MicroRNA: defining a new niche in leukemia. Blood Rev.

[CR86] Finger EC, Cheng CF, Williams TR, Rankin EB, Bedogni B, Tachiki L, Spong S, Giaccia AJ, Powell MB (2014). CTGF is a therapeutic target for metastatic melanoma. Oncogene.

[CR87] Fox JL, Dews M, Minn AJ, Thomas-Tikhonenko A (2013). Targeting of TGFbeta signature and its essential component CTGF by miR-18 correlates with improved survival in glioblastoma. RNA.

[CR88] Frazier K, Williams S, Kothapalli D, Klapper H, Grotendorst GR (1996). Stimulation of fibroblast cell growth, matrix production, and granulation tissue formation by connective tissue growth factor. J Invest Dermatol.

[CR89] Fujita N, Ichii M, Maeda T, Saitoh N, Yokota T, Yamawaki K, Kakitani M, Tomizuka K, Oritani K, Kanakura Y (2015). Identification of osteoblast stimulating factor 5 as a negative regulator in the B-lymphopoietic niche. Exp Hematol.

[CR90] Gale JD, Jensen J, Berman G, Freimuth W, Li G, Pleil A, Kutty M, Rosenthal A, Boswell CB, Noah VEM, Young L (2018). A Placebo-controlled Study of PF-06473871 (anti-connective tissue growth factor antisense oligonucleotide) in reducing hypertrophic skin scarring. Plast Reconstr Surg Glob Open.

[CR91] Gandemer V, Rio AG, de Tayrac M, Sibut V, Mottier S, Ly Sunnaram B, Henry C, Monnier A, Berthou C, Le Gall E, Le Treut A, Schmitt C, Le Gall JY, Mosser J, Galibert MD (2007). Five distinct biological processes and 14 differentially expressed genes characterize TEL/AML1-positive leukemia. BMC Genom.

[CR92] Gao R (2003). Low density lipoprotein receptor-related protein (LRP) is a heparin-dependent adhesion receptor for connective tissue growth factor (CTGF) in rat activated hepatic stellate cells. Hepatol Res.

[CR93] Gao R, Brigstock DR (2004). Connective tissue growth factor (CCN2) induces adhesion of rat activated hepatic stellate cells by binding of its C-terminal domain to integrin alpha(v)beta(3) and heparan sulfate proteoglycan. J Biol Chem.

[CR94] Gao R, Brigstock DR (2005). Connective tissue growth factor (CCN2) in rat pancreatic stellate cell function: integrin alpha5beta1 as a novel CCN2 receptor. Gastroenterology.

[CR95] Gao DF, Niu XL, Hao GH, Peng N, Wei J, Ning N, Wang NP (2007). Rosiglitazone inhibits angiotensin II-induced CTGF expression in vascular smooth muscle cells—role of PPAR-gamma in vascular fibrosis. Biochem Pharmacol.

[CR96] Gerritsen KG, Falke LL, van Vuuren SH, Leeuwis JW, Broekhuizen R, Nguyen TQ, de Borst GJ, Nathoe HM, Verhaar MC, Kok RJ, Goldschmeding R, Visseren FL, Group SS (2016). Plasma CTGF is independently related to an increased risk of cardiovascular events and mortality in patients with atherosclerotic disease: the SMART study. Growth Factors.

[CR97] Gibson DJ, Tuli SS, Schultz GS (2017). Dual-phase iontophoresis for the delivery of antisense oligonucleotides. Nucleic Acid Ther.

[CR98] Gnatenko DV, Dunn JJ, McCorkle SR, Weissmann D, Perrotta PL, Bahou WF (2003). Transcript profiling of human platelets using microarray and serial analysis of gene expression. Blood.

[CR99] Goppelt-Struebe M, Hahn A, Iwanciw D, Rehm M, Banas B (2001). Regulation of connective tissue growth factor (ccn2; ctgf) gene expression in human mesangial cells: modulation by HMG CoA reductase inhibitors (statins). Mol Pathol.

[CR100] Gore-Hyer E, Shegogue D, Markiewicz M, Lo S, Hazen-Martin D, Greene EL, Grotendorst G, Trojanowska M (2002). TGF-beta and CTGF have overlapping and distinct fibrogenic effects on human renal cells. Am J Physiol Renal Physiol.

[CR101] Grobbelaar C, Ford AM (2019). The role of ricroRNA in paediatric acute lymphoblastic leukaemia: challenges for diagnosis and therapy. J Oncol.

[CR102] Grotendorst GR (1997). Connective tissue growth factor: a mediator of TGF-β action on fibroblasts. Cytok Growth Factor Rev.

[CR103] Grotendorst GR, Duncan MR (2005). Individual domains of connective tissue growth factor regulate fibroblast proliferation and myofibroblast differentiation. FASEB J.

[CR104] Grotendorst GR, Okochi H, Hayashi N (1996). A novel transforming growth factor beta response element controls the expression of the connective tissue growth factor gene. Cell Growth Differ.

[CR105] Gu J, Liu X, Wang QX, Tan HW, Guo M, Jiang WF, Zhou L (2012). Angiotensin II increases CTGF expression via MAPKs/TGF-beta1/TRAF6 pathway in atrial fibroblasts. Exp Cell Res.

[CR106] Guerrouahen BS, Al-Hijji I, Tabrizi AR (2011). Osteoblastic and vascular endothelial niches, their control on normal hematopoietic stem cells, and their consequences on the development of leukemia. Stem Cells Int.

[CR107] Guo F, Carter DE, Leask A (2011). Mechanical tension increases CCN2/CTGF expression and proliferation in gingival fibroblasts via a TGFbeta-dependent mechanism. PLoS ONE.

[CR108] Guo Z, Qin Z, Zhang R, Li J, Yin Y (2012). Effect of rosiglitazone on the expression of cardiac adiponectin receptors and NADPH oxidase in type 2 diabetic rats. Eur J Pharmacol.

[CR109] Gupta R, Hong D, Iborra F, Sarno S, Enver T (2007). NOV (CCN3) functions as a regulator of human hematopoietic stem or progenitor cells. Science.

[CR110] Hall-Glenn F, De Young RA, Huang BL, van Handel B, Hofmann JJ, Chen TT, Choi A, Ong JR, Benya PD, Mikkola H, Iruela-Arispe ML, Lyons KM (2012). CCN2/connective tissue growth factor is essential for pericyte adhesion and endothelial basement membrane formation during angiogenesis. PLoS ONE.

[CR111] Hashimoto G, Inoki I, Fujii Y, Aoki T, Ikeda E, Okada Y (2002). Matrix metalloproteinases cleave connective tissue growth factor and reactivate angiogenic activity of vascular endothelial growth factor 165. J Biol Chem.

[CR112] He M, Chen Z, Martin M, Zhang J, Sangwung P, Woo B, Tremoulet AH, Shimizu C, Jain MK, Burns JC, Shyy JY (2017). miR-483 Targeting of CTGF suppresses endothelial-to-mesenchymal transition: therapeutic implications in Kawasaki disease. Circ Res.

[CR113] Heroult M, Bernard-Pierrot I, Delbe J, Hamma-Kourbali Y, Katsoris P, Barritault D, Papadimitriou E, Plouet J, Courty J (2004). Heparin affin regulatory peptide binds to vascular endothelial growth factor (VEGF) and inhibits VEGF-induced angiogenesis. Oncogene.

[CR114] Higgins DF, Biju MP, Akai Y, Wutz A, Johnson RS, Haase VH (2004). Hypoxic induction of Ctgf is directly mediated by Hif-1. Am J Physiol Renal Physiol.

[CR115] Ho YH, Méndez-Ferrer S (2020). Microenvironmental contributions to hematopoietic stem cell aging. Haematologica.

[CR116] Holbourn KP, Acharya KR, Perbal B (2008). The CCN family of proteins: structure-function relationships. Trends Biochem Sci.

[CR117] Holbourn KP, Perbal B, Ravi Acharya K (2009). Proteins on the catwalk: modelling the structural domains of the CCN family of proteins. J Cell Commun Signal.

[CR118] Holmes A, Abraham DJ, Sa S, Shiwen X, Black CM, Leask A (2001). CTGF and SMADs, maintenance of scleroderma phenotype is independent of SMAD signaling. J Biol Chem.

[CR119] Holmes A, Abraham DJ, Chen Y, Denton C, Shi-wen X, Black CM, Leask A (2003). Constitutive connective tissue growth factor expression in scleroderma fibroblasts is dependent on Sp1. J Biol Chem.

[CR120] Honjo T, Kubota S, Kamioka H, Sugawara Y, Ishihara Y, Yamashiro T, Takigawa M, Takano-Yamamoto T (2012). Promotion of Ccn2 expression and osteoblastic differentiation by actin polymerization, which is induced by laminar fluid flow stress. J Cell Commun Signal.

[CR121] Hoshijima M, Hattori T, Inoue M, Araki D, Hanagata H, Miyauchi A, Takigawa M (2006). CT domain of CCN2/CTGF directly interacts with fibronectin and enhances cell adhesion of chondrocytes through integrin alpha5beta1. FEBS Lett.

[CR122] Hoshijima M, Hattori T, Aoyama E, Nishida T, Yamashiro T, Takigawa M (2012). Roles of heterotypic CCN2/CTGF-CCN3/NOV and homotypic CCN2-CCN2 interactions in expression of the differentiated phenotype of chondrocytes. FEBS J.

[CR123] Huang X, Zhu B, Wang X, Xiao R, Wang C (2016). Three-dimensional co-culture of mesenchymal stromal cells and differentiated osteoblasts on human bio-derived bone scaffolds supports active multi-lineage hematopoiesis in vitro: functional implication of the biomimetic HSC niche. Int J Mol Med.

[CR124] Hussein K, Stucki-Koch A, Kreipe H (2018). Profile of fibrosis-related gene transcripts and megakaryocytic changes in the bone marrow of myelodysplastic syndromes with fibrosis. Ann Hematol.

[CR125] Igarashi A, Okochi H, Bradham DM, Grotendorst GR (1993). Regulation of connective tissue growth factor gene expression in human skin fibroblasts and during wound repair. Mol Biol Cell.

[CR126] Igarashi A, Segoshi K, Sakai Y, Pan H, Kanawa M, Higashi Y, Sugiyama M, Nakamura K, Kurihara H, Yamaguchi S, Tsuji K, Kawamoto T, Kato Y (2007). Selection of common markers for bone marrow stromal cells from various bones using real-time RT-PCR: effects of passage number and donor age. Tissue Eng.

[CR127] Ihm SH, Chang K, Kim HY, Baek SH, Youn HJ, Seung KB, Kim JH (2010). Peroxisome proliferator-activated receptor-gamma activation attenuates cardiac fibrosis in type 2 diabetic rats: the effect of rosiglitazone on myocardial expression of receptor for advanced glycation end products and of connective tissue growth factor. Basic Res Cardiol.

[CR128] Inoki I, Shiomi T, Hashimoto G, Enomoto H, Nakamura H, Makino K-i, Ikeda E, Takata S, Kobayashi K-i, Okada Y (2002). Connective tissue growth factor binds vascular endothelial growth factor (VEGF) and inhibits VEGF-induced angiogenesis. FASEB J.

[CR129] Inoue T, Okada H, Kobayashi T, Watanabe Y, Kanno Y, Kopp JB, Nishida T, Takigawa M, Ueno M, Nakamura T, Suzuki H (2003). Hepatocyte growth factor counteracts transforming growth factor-beta1, through attenuation of connective tissue growth factor induction, and prevents renal fibrogenesis in 5/6 nephrectomized mice. FASEB J.

[CR130] Ishihara J, Umemoto T, Yamato M, Shiratsuchi Y, Takaki S, Petrich BG, Nakauchi H, Eto K, Kitamura T, Okano T (2014). Nov/CCN3 regulates long-term repopulating activity of murine hematopoietic stem cells via integrin alphavbeta3. Int J Hematol.

[CR131] Istvanffy R, Vilne B, Schreck C, Ruf F, Pagel C, Grziwok S, Henkel L, Prazeres da Costa O, Berndt J, Stumpflen V, Gotze KS, Schiemann M, Peschel C, Mewes HW, Oostendorp RAJ (2015). Stroma-derived connective tissue growth factor maintains cell cycle progression and repopulation activity of hematopoietic stem cells in vitro stem. Cell Reports.

[CR132] Ito Y, Aten J, Bende RJ, Oemar BS, Rabelink TJ, Weening JJ, Goldschmeding R (1998). Expression of connective tissue growth factor in human renal fibrosis. Kidney Int.

[CR133] Ivkovic S, Yoon BS, Popoff SN, Safadi FF, Libuda DE, Stephenson RC, Daluiski A, Lyons KM (2003). Connective tissue growth factor coordinates chondrogenesis and angiogenesis during skeletal development. Development.

[CR134] Jedsadayanmata A, Chen CC, Kireeva ML, Lau LF, Lam SC (1999). Activation-dependent adhesion of human platelets to Cyr61 and Fisp12/mouse connective tissue growth factor is mediated through integrin alpha(IIb)beta(3). J Biol Chem.

[CR135] Jensen J, Gentzkow G, Berman G, Senne L, Jewell M, Connall TP, Miller SR, Galiano RD, Young L (2018). Anti-CTGF oligonucleotide reduces severity of postsurgical hypertrophic scars in a randomized double-blind, within-subject, placebo-controlled study. Plast Reconstr Surg.

[CR136] Jeon KI, Phipps RP, Sime PJ, Huxlin KR (2015). Inhibitory effects of PPARgamma ligands on TGF-beta1-induced CTGF expression in cat corneal fibroblasts. Exp Eye Res.

[CR137] Ji X, Song L, Sheng L, Gao A, Zhao Y, Han S, Zhang Y, Zhu C, Zhao S, Wang Z, Xu B, Li L, Li J, Tan N, Zhao B (2018). Cyclopeptide RA-V inhibits organ enlargement and tumorigenesis induced by YAP activation. Cancers (Basel).

[CR138] Johnson SK, Stewart JP, Bam R, Qu P, Barlogie B, van Rhee F, Shaughnessy JD, Epstein J, Yaccoby S (2014). CYR61/CCN1 overexpression in the myeloma microenvironment is associated with superior survival and reduced bone disease. Blood.

[CR139] Johnson BG, Ren S, Karaca G, Gomez IG, Fligny C, Smith B, Ergun A, Locke G, Gao B, Hayes S, MacDonnell S, Duffield JS (2017). Connective tissue growth factor domain 4 amplifies fibrotic kidney disease through activation of LDL receptor-related protein 6. J Am Soc Nephrol.

[CR140] Jun JI, Lau LF (2011). Taking aim at the extracellular matrix: CCN proteins as emerging therapeutic targets. Nat Rev Drug Discov.

[CR141] Jung Y, Wang J, Schneider A, Sun YX, Koh-Paige AJ, Osman NI, McCauley LK, Taichman RS (2006). Regulation of SDF-1 (CXCL12) production by osteoblasts; a possible mechanism for stem cell homing. Bone.

[CR142] Kaasboll OJ, Gadicherla AK, Wang JH, Monsen VT, Hagelin EMV, Dong MQ, Attramadal H (2018). Connective tissue growth factor (CCN2) is a matricellular preproprotein controlled by proteolytic activation. J Biol Chem.

[CR143] Kafi R, Fisher GJ, Quan T, Shao Y, Wang R, Voorhees JJ, Kang S (2004). UV-A1 phototherapy improves nephrogenic fibrosing dermopathy. Arch Dermatol.

[CR144] Kang H, Chen IM, Wilson CS, Bedrick EJ, Harvey RC, Atlas SR, Devidas M, Mullighan CG, Wang X, Murphy M, Ar K, Wharton W, Borowitz MJ, Bowman WP, Bhojwani D, Carroll WL, Camitta BM, Reaman GH, Smith MA, Downing JR, Hunger SP, Willman CL (2010). Gene expression classifiers for relapse-free survival and minimal residual disease improve risk classification and outcome prediction in pediatric B-precursor acute lymphoblastic leukemia. Blood.

[CR145] Kang S, Kim J, Ahn M, Kim J, Heo MG, Min DH, Won C (2020). RNAi nanotherapy for fibrosis: highly durable knockdown of CTGF/CCN-2 using siRNA-DegradaBALL (LEM-S401) to treat skin fibrotic diseases. Nanoscale.

[CR146] Kanyama M, Kuboki T, Akiyama K, Nawachi K, Miyauchi FM, Yatani H, Kubota S, Nakanishi T, Takigawa M (2003). Connective tissue growth factor expressed in rat alveolar bone regeneration sites after tooth extraction. Arch Oral Biol.

[CR147] Kawaki H, Kubota S, Suzuki A, Lazar N, Yamada T, Matsumura T, Ohgawara T, Maeda T, Perbal B, Lyons KM, Takigawa M (2008). Cooperative regulation of chondrocyte differentiation by CCN2 and CCN3 shown by a comprehensive analysis of the CCN family proteins in cartilage. J Bone Miner Res.

[CR148] Kawata K, Eguchi T, Kubota S, Kawaki H, Oka M, Minagi S, Takigawa M (2006). Possible role of LRP1, a CCN2 receptor, in chondrocytes. Biochem Biophys Res Commun.

[CR149] Kawata K, Kubota S, Eguchi T, Aoyama E, Moritani NH, Kondo S, Nishida T, Takigawa M (2012). Role of LRP1 in transport of CCN2 protein in chondrocytes. J Cell Sci.

[CR150] Kemp TJ, Aggeli IK, Sugden PH, Clerk A (2004). Phenylephrine and endothelin-1 upregulate connective tissue growth factor in neonatal rat cardiac myocytes. J Mol Cell Cardiol.

[CR151] Kessler D, Dethlefsen S, Haase I, Plomann M, Hirche F, Krieg T, Eckes B (2001). Fibroblasts in mechanically stressed collagen lattices assume a "synthetic" phenotype. J Biol Chem.

[CR152] Khankan R, Oliver N, He S, Ryan SJ, Hinton DR (2011). Regulation of fibronectin-EDA through CTGF domain-specific interactions with TGFβ2 and its receptor TGFβRII. Invest Ophthalmol Vis Sci.

[CR153] Khattab HM, Aoyama E, Kubota S, Takigawa M (2015). Physical interaction of CCN2 with diverse growth factors involved in chondrocyte differentiation during endochondral ossification. J Cell Commun Signal.

[CR154] Kim HS, Nagalla SR, Oh Y, Wilson E, Roberts CT, Rosenfeld RG (1997). Identification of a family of low-affinity insulin-like growth factor binding proteins (IGFBPs): characterization of connective tissue growth factor as a member of the IGFBP superfamily. Proc Natl Acad Sci.

[CR155] Kimura A, Martin C, Robinson GW, Simone JM, Chen W, Wickre MC, O'Shea JJ, Hennighausen L (2010). The gene encoding the hematopoietic stem cell regulator CCN3/NOV is under direct cytokine control through the transcription factors STAT5A/B. J Biol Chem.

[CR156] Kinashi H, Falke LL, Nguyen TQ, Bovenschen N, Aten J, Leask A, Ito Y, Goldschmeding R (2017). Connective tissue growth factor regulates fibrosis-associated renal lymphangiogenesis. Kidney Int.

[CR157] Kireeva ML, Latinkić BV, Yang GP, Abler AS, Kolesnikova TV, Chen C-C, Lau LF (1997). Cyr61 and Fisp12 are both ECM-associated signaling molecules: activities, metabolism, and localization during development. Exp Cell Res.

[CR158] Kiwanuka E, Andersson L, Caterson EJ, Junker JP, Gerdin B, Eriksson E (2013). CCN2 promotes keratinocyte adhesion and migration via integrin alpha5beta1. Exp Cell Res.

[CR159] Klamer S, Voermans C (2014). The role of novel and known extracellular matrix and adhesion molecules in the homeostatic and regenerative bone marrow microenvironment. Cell Adhes Migr.

[CR160] Ko WC, Chen BC, Hsu MJ, Tsai CT, Hong CY, Lin CH (2012). Thrombin induced connective tissue growth factor expression in rat vascular smooth muscle cells via the PAR-1/JNK/AP-1 pathway. Acta Pharmacol Sin.

[CR161] Koitabashi N, Arai M, Niwano K, Watanabe A, Endoh M, Suguta M, Yokoyama T, Tada H, Toyama T, Adachi H, Naito S, Oshima S, Nishida T, Kubota S, Takigawa M, Kurabayashi M (2008). Plasma connective tissue growth factor is a novel potential biomarker of cardiac dysfunction in patients with chronic heart failure. Eur J Heart Fail.

[CR162] Kondo S, Kubota S, Shimo T, Nishida T, Yosimichi G, Eguchi T, Sugahara T, Takigawa M (2002). Connective tissue growth factor increased by hypoxia may initiate angiogenesis in collaboration with matrix metalloproteinases. Carcinogenesis.

[CR163] Kondo S, Tanaka N, Kubota S, Mukudai Y, Yosimichi G, Sugahara T, Takigawa M (2006). Novel angiogenic inhibitor DN-9693 that inhibits post-transcriptional induction of connective tissue growth factor (CTGF/CCN2) by vascular endothelial growth factor in human endothelial cells. Mol Cancer Ther.

[CR164] Konopleva M, Mikhail A, Estrov Z, Zhao S, Harris D, Sanchez-Williams G, Kornblau SM, Dong J, Kliche K-O, Jiang S, Snodgrass HR, Estey EH, Andreeff M (1999). Expression and function of leptin receptor isoforms in myeloid leukemia and myelodysplastic syndromes: proliferative and anti-apoptotic activities. Blood.

[CR165] Koralov SB, Muljo SA, Galler GR, Krek A, Chakraborty T, Kanellopoulou C, Jensen K, Cobb BS, Merkenschlager M, Rajewsky N, Rajewsky K (2008). Dicer ablation affects antibody diversity and cell survival in the B lymphocyte lineage. Cell.

[CR166] Korn C, Mendez-Ferrer S (2017). Myeloid malignancies and the microenvironment. Blood.

[CR167] Kothapalli D, Hayashi N, Grotendorst GR (1998). Inhibition of TGF-beta-stimulated CTGF gene expression and anchorage-independent growth by cAMP identifies a CTGF-dependent restriction point in the cell cycle. FASEB J.

[CR168] Kroening S, Solomovitch S, Sachs M, Wullich B, Goppelt-Struebe M (2009). Regulation of connective tissue growth factor (CTGF) by hepatocyte growth factor in human tubular epithelial cells. Nephrol Dial Transplant.

[CR169] Kubota S, Takigawa M (2013). The CCN family acting throughout the body: recent research developments. Biomol Concepts.

[CR170] Kubota S, Moritani NH, Kawaki H, Mimura H, Minato M, Takigawa M (2003). Transcriptional induction of connective tissue growth factor/hypertrophic chondrocyte-specific 24 gene by dexamethasone in human chondrocytic cells. Bone.

[CR171] Kubota S, Kawata K, Yanagita T, Doi H, Kitoh T, Takigawa M (2004). Abundant retention and release of connective tissue growth factor (CTGF/CCN2) by platelets. J Biochem.

[CR172] Kubota S, Kawaki H, Kondo S, Yosimichi G, Minato M, Nishida T, Hanagata H, Miyauchi A, Takigawa M (2006). Multiple activation of mitogen-activated protein kinases by purified independent CCN2 modules in vascular endothelial cells and chondrocytes in culture. Biochimie.

[CR173] Lachowski D, Cortes E, Robinson B, Rice A, Rombouts K, Del Rio Hernandez AE (2018). FAK controls the mechanical activation of YAP, a transcriptional regulator required for durotaxis. FASEB J.

[CR174] Lam S, van der Geest RN, Verhagen NAM, van Nieuwenhoven FA, Blom IE, Aten J, Goldschmeding R, Daha MR, van Kooten C (2003). Connective tissue growth factor and igf-I are produced by human renal fibroblasts and cooperate in the induction of collagen production by high glucose. Diabetes.

[CR175] Lambi AG, Pankratz TL, Mundy C, Gannon M, Barbe MF, Richtsmeier JT, Popoff SN (2012). The skeletal site-specific role of connective tissue growth factor in prenatal osteogenesis. Dev Dyn.

[CR176] Lau LF (2016). Cell surface receptors for CCN proteins. J Cell Commun Signal.

[CR177] Lau LF, Lam SC-T (1999). The CCN family of angiogenic regulators: The integrin connection. Ex Cell Res.

[CR178] Laug R, Fehrholz M, Schutze N, Kramer BW, Krump-Konvalinkova V, Speer CP, Kunzmann S (2012). IFN-gamma and TNF-alpha synergize to inhibit CTGF expression in human lung endothelial cells. PLoS ONE.

[CR179] Lazzari E, Butler JM (2018). The instructive role of the bone marrow niche in aging and leukemia. Curr Stem Cell Rep.

[CR180] Leask A (2020). Conjunction junction, what's the function? CCN proteins as targets in fibrosis and cancers. Am J Physiol Cell Physiol.

[CR181] Leask A, Parapuram SK, Shi-Wen X, Abraham DJ (2009). Connective tissue growth factor (CTGF, CCN2) gene regulation: a potent clinical bio-marker of fibroproliferative disease?. J Cell Commun Signal.

[CR182] Lee Y-S, Kim J-A, Kim KL, Jang H-S, Kim J-M, Lee J-Y, Shin I-S, Lee S-J, Suh W, Choi J-H, Jeon E-S, Byun J, Kim D-K (2004). Aldosterone upregulates connective tissue growth factor gene expression via p38 MAPK pathway and mineralocorticoid receptor in ventricular myocytes. J Korean Med Sci.

[CR183] Lee CH, Shah B, Moioli EK, Mao JJ (2010). CTGF directs fibroblast differentiation from human mesenchymal stem/stromal cells and defines connective tissue healing in a rodent injury model. J Clin Invest.

[CR184] Lee MS, Ghim J, Kim SJ, Yun YS, Yoo SA, Suh PG, Kim WU, Ryu SH (2015). Functional interaction between CTGF and FPRL1 regulates VEGF-A-induced angiogenesis. Cell Signal.

[CR185] Li AJ, Calvi LM (2017). The microenvironment in myelodysplastic syndromes: Niche-mediated disease initiation and progression. Exp Hematol.

[CR186] Li G, Xie Q, Shi Y, Li D, Zhang M, Jiang S, Zhou H, Lu H, Jin Y (2006). Inhibition of connective tissue growth factor by siRNA prevents liver fibrosis in rats. J Gene Med.

[CR187] Li X, Ling W, Khan S, Yaccoby S (2012). Therapeutic effects of intrabone and systemic mesenchymal stem cell cytotherapy on myeloma bone disease and tumor growth. J Bone Miner Res.

[CR188] Li H, Li J, Cheng J, Chen X, Zhou L, Li Z (2019). AMLderived mesenchymal stem cells upregulate CTGF expression through the BMP pathway and induce K562ADM fusiform transformation and chemoresistance. Oncol Rep.

[CR189] Lin J, Liliensiek B, Kanitz M, Schimanski U, Böhrer H, Waldherr R, Martin E, Kauffmann G, Ziegler R, Nawroth PP (1998). Molecular cloning of genes differentially regulated by TNF-alpha in bovine aortic endothelial cells, fibroblasts and smooth muscle cells. Cardiovasc Res.

[CR190] Lindner U, Kramer J, Rohwedel J, Schlenke P (2010). Mesenchymal stem or stromal cells: toward a better understanding of their biology?. Transfus Med Hemother.

[CR191] Liu S, Leask A (2013). CCN2 modulates hair follicle cycling in mice. Mol Biol Cell.

[CR192] Liu X, Luo F, Li J, Wu W, Li L, Chen H (2008). Homocysteine induces connective tissue growth factor expression in vascular smooth muscle cells. J Thromb Haemost.

[CR193] Liu SC, Hsu CJ, Chen HT, Tsou HK, Chuang SM, Tang CH (2012). CTGF increases IL-6 expression in human synovial fibroblasts through integrin-dependent signaling pathway. PLoS ONE.

[CR194] Liu S, Herault Y, Pavlovic G, Leask A (2014). Skin progenitor cells contribute to bleomycin-induced skin fibrosis Arthritis. Rheumatol.

[CR195] Liu H, Peng F, Liu Z, Jiang F, Li L, Gao S, Wang G, Song J, Ruan E, Shao Z, Fu R (2017). CYR61/CCN1 stimulates proliferation and differentiation of osteoblasts in vitro and contributes to bone remodeling in vivo in myeloma bone disease. Int J Oncol.

[CR196] Long X, Yu Y, Perlaky L, Man TK, Redell MS (2015). Stromal CYR61 confers resistance to mitoxantrone via spleen tyrosine kinase activation in human acute myeloid leukaemia. Br J Haematol.

[CR197] Lu H, Kojima K, Battula VL, Korchin B, Shi Y, Chen Y, Spong S, Thomas DA, Kantarjian H, Lock RB, Andreeff M, Konopleva M (2014). Targeting connective tissue growth factor (CTGF) in acute lymphoblastic leukemia preclinical models: anti-CTGF monoclonal antibody attenuates leukemia growth. Ann Hematol.

[CR198] Luo Q, Kang Q, Si W, Jiang W, Park JK, Peng Y, Li X, Luu HH, Luo J, Montag AG, Haydon RC, He TC (2004). Connective tissue growth factor (CTGF) is regulated by Wnt and bone morphogenetic proteins signaling in osteoblast differentiation of mesenchymal stem cells. J Biol Chem.

[CR199] Maeda A, Nishida T, Aoyama E, Kubota S, Lyons KM, Kuboki T, Takigawa M (2009). CCN family 2/connective tissue growth factor modulates BMP signalling as a signal conductor, which action regulates the proliferation and differentiation of chondrocytes. J Biochem.

[CR200] Makino K, Makino T, Stawski L, Lipson KE, Leask A, Trojanowska M (2017). Anti-connective tissue growth factor (CTGF/CCN2) monoclonal antibody attenuates skin fibrosis in mice models of systemic sclerosis. Arthritis Res Ther.

[CR201] Masuko K, Murata M, Yudoh K, Shimizu H, Beppu M, Nakamura H, Kato T (2010). Prostaglandin E2 regulates the expression of connective tissue growth factor (CTGF/CCN2) in human osteoarthritic chondrocytes via the EP4 receptor. BMC Res Notes.

[CR202] McCallum L, Price S, Planque N, Perbal B, Pierce A, Whetton AD, Irvine AE (2006). A novel mechanism for BCR-ABL action: stimulated secretion of CCN3 is involved in growth and differentiation regulation. Blood.

[CR203] McCallum L, Lu W, Price S, Lazar N, Perbal B, Irvine AE (2009). CCN3: a key growth regulator in chronic myeloid leukaemia. J Cell Commun Signal.

[CR204] McCallum L, Lu W, Price S, Lazar N, Perbal B, Irvine AE (2012). CCN3 suppresses mitogenic signalling and reinstates growth control mechanisms in chronic myeloid leukaemia. J Cell Commun Signal.

[CR205] Mercurio S, Latinkic B, Itasaki N, Krumlauf R, Smith JC (2004). Connective-tissue growth factor modulates WNT signalling and interacts with the WNT receptor complex. Development.

[CR206] Midwood KS, Williams LV, Schwarzbauer JE (2004). Tissue repair and the dynamics of the extracellular matrix. Int J Biochem Cell Biol.

[CR207] Miyazaki O, Kurashita S, Fukamachi I, Endo K, Ng PS, Takehara K (2010). Subtraction method for determination of N-terminal connective tissue growth factor. Ann Clin Biochem.

[CR208] Möhle R, Marcus Schittenhelm M, Failenschmid C, Bautz F, Kratz-Albers K, Serve H, Brugger W, Lothar Kanz L (2000). Functional response of leukaemic blasts to stromal cell-derived factor-1 correlates with preferential expression of the chemokine receptor CXCR4 in acute myelomonocytic and lymphoblastic leukaemia. Br J Haematol.

[CR209] Mokalled MH, Patra C, Dickson AL, Endo T, Stainier DY, Poss KD (2016). Injury-induced ctgfa directs glial bridging and spinal cord regeneration in zebrafish. Science.

[CR210] Moran-Jones K, Gloss BS, Murali R, Chang DK, Colvin EK, Jones MD, Yuen S, Howell VM, Brown LM, Wong CW, Sprong SM, Scarlett CJ, Hacker NF, Ghosh S, Mok SC, Birrer MJ, Samimi G (2015). Connective tissue growth factor as a novel therapeutic target in high grade serous ovarian cancer. Oncotarget.

[CR211] Mori T, Kawara A, Shinozaki M, Hayashi N, Kakinuma T, Igarashi A, Takigawa M, Nakanishi T, Takehara K (1999). Role and interaction of connective tissue growth factor with transforming growth factor-beta in persistent fibrosis: a mouse fibrosis model. J Cell Physiol.

[CR212] Mori Y, Hinchcliff M, Wu M, Warner-Blankenship M, Lyons KM, Varga J (2008). Connective tissue growth factor/CCN2-null mouse embryonic fibroblasts retain intact transforming growth factor-beta responsiveness. Exp Cell Res.

[CR213] Moritani NH, Kubota S, Sugahara T, Takigawa M (2005). Comparable response of ccn1 with ccn2 genes upon arthritis: An in vitro evaluation with a human chondrocytic cell line stimulated by a set of cytokines. Cell Commun Signal.

[CR214] Morrison SJ, Scadden DT (2014). The bone marrow niche for haematopoietic stem cells. Nature.

[CR215] Morrison SJ, Spradling AC (2008). Stem cells and niches: mechanisms that promote stem cell maintenance throughout life. Cell.

[CR216] Mu S, Kang B, Zeng W, Sun Y, Yang F (2016). MicroRNA-143–3p inhibits hyperplastic scar formation by targeting connective tissue growth factor CTGF/CCN2 via the Akt/mTOR pathway. Mol Cell Biochem.

[CR217] Muehlich S, Schneider N, Hinkmann F, Garlichs CD, Goppelt-Struebe M (2004). Induction of connective tissue growth factor (CTGF) in human endothelial cells by lysophosphatidic acid, sphingosine-1-phosphate, and platelets. Atherosclerosis.

[CR218] Mundy C, Gannon M, Popoff SN (2014). Connective tissue growth factor (CTGF/CCN2) negatively regulates BMP-2 induced osteoblast differentiation and signaling. J Cell Physiol.

[CR219] Munemasa S, Sakai A, Kuroda Y, Okikawa Y, Katayama Y, Asaoku H, Kubo T, Miyakawa Y, Serikawa M, Sasaki T, Kimura A (2007). Connective tissue growth factor is an indicator of bone involvement in multiple myeloma, but matrix metalloproteinase-9 is not. Br J Haematol.

[CR220] Murphy M, Godson C, Cannon S, Kato S, Mackenzie HS, Martin F, Brady HR (1999). Suppression subtractive hybridization identifies high glucose levels as a stimulus for expression of connective tissue growth factor and other genes in human mesangial cells. J Biol Chem.

[CR221] Murphy-Ullrich JE, Sage EH (2014). Revisiting the matricellular concept. Matrix Biol.

[CR222] Nagasawa-Masuda A, Terai K (2017). Yap/Taz transcriptional activity is essential for vascular regression via Ctgf expression and actin polymerization. PLoS ONE.

[CR223] Nakanishi T, Kimura Y, Tamura T, Ichikawa H, Yamaai YI, Sugimoto T, Takigawa M (1997). Cloning of a mRNA preferentially expressed in chondrocytes by differential display-PCR from a human chondrocytic cell line that is identical with connective tissue growth factor (CTGF) mRNA. Biochem Biophys Res Commun.

[CR224] Naveiras O, Nardi V, Wenzel PL, Hauschka PV, Fahey F, Daley GQ (2009). Bone-marrow adipocytes as negative regulators of the haematopoietic microenvironment. Nature.

[CR225] Neesse A, Frese KK, Bapiro TE, Nakagawa T, Sternlicht MD, Seeley TW, Pilarsky C, Jodrell DI, Spong SM, Tuveson DA (2013). CTGF antagonism with mAb FG-3019 enhances chemotherapy response without increasing drug delivery in murine ductal pancreas cancer. Proc Natl Acad Sci U S A.

[CR226] Neve A, Corrado A, Cantatore FP (2011). Osteoblast physiology in normal and pathological conditions. Cell Tissue Res.

[CR227] Nguyen TQ, Roestenberg P, van Nieuwenhoven FA, Bovenschen N, Li Z, Xu L, Oliver N, Aten J, Joles JA, Vial C, Brandan E, Lyons KM, Goldschmeding R (2008). CTGF inhibits BMP-7 signaling in diabetic nephropathy. J Am Soc Nephrol.

[CR228] Nguyen XX, Muhammad L, Nietert PJ, Feghali-Bostwick C (2018). IGFBP-5 promotes fibrosis via increasing its own expression and that of other pro-fibrotic mediators. Front Endocrinol (Lausanne).

[CR229] Nishida T, Nakanishi T, Shimo T, Takigawa M (2000). Effects of CTGF/Hcs24, a hypertrophic chondrocyte-specific gene product, on the proliferation and differentiation of osteoblastic cells in vitro. J Cell Physiol.

[CR230] Nishida T, Kubota S, Fukunaga T, Kondo S, Yosimichi G, Nakanishi T, Takano-Yamamoto T, Takigawa M (2003). CTGF/Hcs24, hypertrophic chondrocyte-specific gene product, interacts with perlecan in regulating the proliferation and differentiation of chondrocytes. J Cell Physiol.

[CR231] Nishida T, Kubota S, Kojima S, Kuboki T, Nakao K, Kushibiki T, Tabata Y, Takigawa M (2004). Regeneration of defects in articular cartilage in rat knee joints by CCN2 (connective tissue growth factor). J Bone Miner Res.

[CR232] Nishida T, Emura K, Kubota S, Lyons KM, Takigawa M (2011). CCN family 2/connective tissue growth factor (CCN2/CTGF) promotes osteoclastogenesis via induction of and interaction with dendritic cell-specific transmembrane protein (DC-STAMP). J Bone Miner Res.

[CR233] Nishida T, Kubota S, Aoyama E, Janune D, Maeda A, Takigawa M (2011). Effect of CCN2 on FGF2-induced proliferation and MMP9 and MMP13 productions by chondrocytes. Endocrinology.

[CR234] Niu CC, Zhao C, Yang Z, Zhang XL, Pan J, Zhao C, Si WK (2014). Inhibiting CCN1 blocks AML cell growth by disrupting the MEK/ERK pathway. Cancer Cell Int.

[CR235] Ohara Y, Chew SH, Misawa N, Wang S, Somiya D, Nakamura K, Kajiyama H, Kikkawa F, Tsuyuki Y, Jiang L, Yamashita K, Sekido Y, Lipson KE, Toyokuni S (2018). Connective tissue growth factor-specific monoclonal antibody inhibits growth of malignant mesothelioma in an orthotopic mouse model. Oncotarget.

[CR236] Ohkawara B, Kobayakawa A, Kanbara S, Hattori T, Kubota S, Ito M, Masuda A, Takigawa M, Lyons KM, Ishiguro N, Ohno K (2020). CTGF/CCN2 facilitates LRP4-mediated formation of the embryonic neuromuscular junction. EMBO Rep.

[CR237] Ohnishi H, Oka T, Kusachi S, Nakanishi T, Takeda K, Nakahama M, Doi M, Murakami T, Ninomiya Y, Takigawa M, Tsuji T (1998). Increased expression of connective tissue growth factor in the infarct zone of experimentally induced myocardial infarction in rats. J Mol Cell Cardiol.

[CR238] Okada H, Kikuta T, Kobayashi T, Inoue T, Kanno Y, Takigawa M, Sugaya T, Kopp JB, Suzuki H (2005). Connective tissue growth factor expressed in tubular epithelium plays a pivotal role in renal fibrogenesis. J Am Soc Nephrol.

[CR239] Okada H, Kikuta T, Inoue T, Kanno Y, Ban S, Sugaya T, Takigawa M, Suzuki H (2006). Dexamethasone induces connective tissue growth factor expression in renal tubular epithelial cells in a mouse strain-specific manner. Am J Pathol.

[CR240] Omoto S, Nishida K, Yamaai Y, Shibahara M, Nishida T, Doi T, Asahara H, Nakanishi T, Inoue H, Takigawa M (2004). Expression and localization of connective tissue growth factor (CTGF/Hcs24/CCN2) in osteoarthritic cartilage. Osteoarthr Cartil.

[CR241] Pan LH, Yamauchi K, Uzuki M, Nakanishi T, Takigawa M, Inoue H, Sawai T (2001). Type II alveolar epithelial cells and interstitial fibroblasts express connective tissue growth factor in IPF. Eur Respir J.

[CR242] Parada C, Li J, Iwata J, Suzuki A, Chai Y (2013). CTGF mediates Smad-dependent transforming growth factor beta signaling to regulate mesenchymal cell proliferation during palate development. Mol Cell Biol.

[CR243] Paradis V, Perlemuter G, Bonvoust F, Dargere D, Parfait B, Vidaud M, Conti M, Huet S, Ba N, Buffet C, Bedossa P (2001). High glucose and hyperinsulinemia stimulate connective tissue growth factor expression: a potential mechanism involved in progression to fibrosis in nonalcoholic steatohepatitis. Hepatology.

[CR244] Peidl A, Perbal B, Leask A (2019). Yin/Yang expression of CCN family members: Transforming growth factor beta 1, via ALK5/FAK/MEK, induces CCN1 and CCN2, yet suppresses CCN3, expression in human dermal fibroblasts. PLoS ONE.

[CR245] Perbal B (2018). The concept of the CCN protein family revisited: a centralized coordination network. J Cell Commun Signal.

[CR246] Perbal B, Tweedie S, Bruford E (2018). The official unified nomenclature adopted by the HGNC calls for the use of the acronyms, CCN1–6, and discontinuation in the use of CYR61, CTGF, NOV and WISP 1–3 respectively. J Cell Commun Signal.

[CR247] Pereira RC, Durant D, Canalis E (2000). Transcriptional regulation of connective tissue growth factor by cortisol in osteoblasts. Am J Physiol Endor Metab.

[CR248] Pi L, Ding X, Jorgensen M, Pan JJ, Oh SH, Pintilie D, Brown A, Song WY, Petersen BE (2008). Connective tissue growth factor with a novel fibronectin binding site promotes cell adhesion and migration during rat oval cell activation. Hepatology.

[CR249] Pi L, Shenoy AK, Liu J, Kim S, Nelson N, Xia H, Hauswirth WW, Petersen BE, Schultz GS, Scott EW (2012). CCN2/CTGF regulates neovessel formation via targeting structurally conserved cystine knot motifs in multiple angiogenic regulators. FASEB J.

[CR250] Pinho S, Frenette PS (2019). Haematopoietic stem cell activity and interactions with the niche. Nat Rev Mol Cell Biol.

[CR251] Piszczatowski RT, Rafferty BJ, Rozado A, Parziale JV, Lents NH (2015). Myeloid Zinc Finger 1 (MZF-1) regulates expression of the CCN2/CTGF and CCN3/NOV genes in the hematopoietic compartment. J Cell Physiol.

[CR252] Pobbati AV, Hong W (2020). A combat with the YAP/TAZ-TEAD oncoproteins for cancer therapy. Theranostics.

[CR253] Preisser F, Giehl K, Rehm M, Goppelt-Struebe M (2016). Inhibitors of oxygen sensing prolyl hydroxylases regulate nuclear localization of the transcription factors Smad2 and YAP/TAZ involved in CTGF synthesis. Biochim Biophys Acta.

[CR254] Qiao G, Xia D, Cheng Z, Zhang G (2017). miR132 in atrial fibrillation directly targets connective tissue growth factor. Mol Med Rep.

[CR255] Quan T, Shin S, Qin Z, Fisher GJ (2009). Expression of CCN family of genes in human skin in vivo and alterations by solar-simulated ultraviolet irradiation. J Cell Commun Signal.

[CR256] Raghu G, Scholand MB, de Andrade J, Lancaster L, Mageto Y, Goldin J, Brown KK, Flaherty KR, Wencel M, Wanger J, Neff T, Valone F, Stauffer J, Porter S (2016). FG-3019 anti-connective tissue growth factor monoclonal antibody: results of an open-label clinical trial in idiopathic pulmonary fibrosis. Eur Respir J.

[CR257] Raghunathan VK, Dreier B, Morgan JT, Tuyen BC, Rose BW, Reilly CM, Russell P, Murphy CJ (2014). Involvement of YAP, TAZ and HSP90 in contact guidance and intercellular junction formation in corneal epithelial cells. PLoS ONE.

[CR258] Ramazani Y, Knops N, Elmonem MA, Nguyen TQ, Arcolino FO, van den Heuvel L, Levtchenko E, Kuypers D, Goldschmeding R (2018). Connective tissue growth factor (CTGF) from basics to clinics. Matrix Biol.

[CR259] Rayego-Mateos S, Rodrigues-Diez R, Morgado-Pascual JL, Rodrigues Diez RR, Mas S, Lavoz C, Alique M, Pato J, Keri G, Ortiz A, Egido J, Ruiz-Ortega M (2013). Connective tissue growth factor is a new ligand of epidermal growth factor receptor. J Mol Cell Biol.

[CR260] Ren J, Jin P, Sabatino M, Balakumaran A, Feng J, Kuznetsov SA, Klein HG, Robey PG, Stroncek DF (2011). Global transcriptome analysis of human bone marrow stromal cells (BMSC) reveals proliferative, mobile and interactive cells that produce abundant extracellular matrix proteins, some of which may affect BMSC potency. Cytotherapy.

[CR261] Ren S, Johnson BG, Kida Y, Ip C, Davidson KC, Lin S-L, Kobayashi A, Lang RA, Hadjantonakis A-K, Moon RT, Duffield JS (2013). LRP-6 is a coreceptor for multiple fibrogenic signaling pathways in pericytes and myofibroblasts that are inhibited by DKK-1. Proc Natl Acad Sci U S A.

[CR262] Ren Y, Du C, Shi Y, Wei J, Wu H, Cui H (2017). The Sirt1 activator, SRT1720, attenuates renal fibrosis by inhibiting CTGF and oxidative stress. Int J Mol Med.

[CR263] Richeldi L, Fernández Pérez ER, Costabel U, Albera C, Lederer DJ, Flaherty KR, Ettinger N, Perez R, Scholand MB, Goldin J, Peony Yu K-H, Neff T, Porter S, Zhong M, Gorina E, Kouchakji E, Raghu G (2020). Pamrevlumab, an anti-connective tissue growth factor therapy, for idiopathic pulmonary fibrosis (PRAISE): a phase 2, randomised, double-blind, placebo-controlled trial. Lancet Respir Med.

[CR264] Ricupero DA, Rishikof DC, Kuang PP, Poliks CF, Goldstein RH (1999). Regulation of connective tissue growth factor expression by prostaglandin E(2). Am J Physiol.

[CR265] Riser BL, Denichilo M, Cortes P, Baker C, Grondin JM, Yee J, Narins RG (2000). Regulation of connective tissue growth factor activity in cultured rat mesangial cells and its expression in experimental diabetic glomerulosclerosis. J Am Soc Nephrol.

[CR266] Riser BL, Najmabadi F, Perbal B, Peterson DR, Rambow JA, Riser ML, Sukowski E, Yeger H, Riser SC (2009). CCN3 (NOV) is a negative regulator of CCN2 (CTGF) and a novel endogenous inhibitor of the fibrotic pathway in an in vitro model of renal disease. Am J Pathol.

[CR267] Riser BL, Najmabadi F, Perbal B, Rambow JA, Riser ML, Sukowski E, Yeger H, Riser SC, Peterson DR (2010). CCN3/CCN2 regulation and the fibrosis of diabetic renal disease. J Cell Commun Signal.

[CR268] Riser BL, Najmabadi F, Garchow K, Barnes JL, Peterson DR, Sukowski EJ (2014). Treatment with the matricellular protein CCN3 blocks and/or reverses fibrosis development in obesity with diabetic nephropathy. Am J Pathol.

[CR269] Rodriguez-Vita J, Ruiz-Ortega M, Ruperez M, Esteban V, Sanchez-Lopez E, Plaza JJ, Egido J (2005). Endothelin-1, via ETA receptor and independently of transforming growth factor-beta, increases the connective tissue growth factor in vascular smooth muscle cells. Circ Res.

[CR270] Roestenberg P, van Nieuwenhoven FA, Wieten L, Boer P, Diekman T, Tiller AM, Wiersinga WM, Oliver N, Usinger W, Weitz S, Schlingemann RO, Goldschmeding R (2004). Connective tissue growth factor is increased in plasma of type 1 diabetic patients with nephropathy. Diabetes Care.

[CR271] Rooney B, O'Donovan H, Gaffney A, Browne M, Faherty N, Curran SP, Sadlier D, Godson C, Brazil DP, Crean J (2011). CTGF/CCN2 activates canonical Wnt signalling in mesangial cells through LRP6: implications for the pathogenesis of diabetic nephropathy. FEBS Lett.

[CR272] Rozado A, Piszczatowski RT, Rafferty BJ, Lents NH (2014). Regulation of CCN2 and CCN3 in bone marrow through myloid zinc finger-1 and its medical implication in hematopoiesis (1005.4). FASEB J.

[CR273] Sacchetti B, Funari A, Michienzi S, Di Cesare S, Piersanti S, Saggio I, Tagliafico E, Ferrari S, Robey PG, Riminucci M, Bianco P (2007). Self-renewing osteoprogenitors in bone marrow sinusoids can organize a hematopoietic microenvironment. Cell.

[CR274] Safadi FF, Xu J, Smock SL, Kanaan RA, Selim AH, Odgren PR, Marks SC, Owen TA, Popoff SN (2003). Expression of connective tissue growth factor in bone: its role in osteoblast proliferation and differentiation in vitro and bone formation in vivo. J Cell Physiol.

[CR275] Sakai N, Nakamura M, Lipson KE, Miyake T, Kamikawa Y, Sagara A, Shinozaki Y, Kitajima S, Toyama T, Hara A, Iwata Y, Shimizu M, Furuichi K, Kaneko S, Tager AM, Wada T (2017). Inhibition of CTGF ameliorates peritoneal fibrosis through suppression of fibroblast and myofibroblast accumulation and angiogenesis. Sci Rep.

[CR276] Sala-Torra O, Gundacker HM, Stirewalt DL, Ladne PA, Pogosova-Agadjanyan EL, Slovak ML, Willman CL, Heimfeld S, Boldt DH, Radich JP (2007). Connective tissue growth factor (CTGF) expression and outcome in adult patients with acute lymphoblastic leukemia. Blood.

[CR277] Sangaletti S, Chiodoni C, Tripodo C, Colombo MP (2017). Common extracellular matrix regulation of myeloid cell activity in the bone marrow and tumor microenvironments. Cancer Immunol Immunother.

[CR278] Schepers K, Pietras EM, Reynaud D, Flach J, Binnewies M, Garg T, Wagers AJ, Hsiao EC, Passegue E (2013). Myeloproliferative neoplasia remodels the endosteal bone marrow niche into a self-reinforcing leukemic niche. Cell Stem Cell.

[CR279] Schild C, Trueb B (2002). Mechanical stress is required for high-level expression of connective tissue growth factor. Exp Cell Res.

[CR280] Schild C, Trueb B (2004). Three members of the connective tissue growth factor family CCN are differentially regulated by mechanical stress. Biochim Biophys Acta.

[CR281] Schober JM, Chen N, Grzeszkiewicz TM, Jovanovic I, Emeson EE, Ugarova TP, Ye RD, Lau LF, Lam SC-T (2002). Identification of integrin alpha(M)beta(2) as an adhesion receptor on peripheral blood monocytes for Cyr61 (CCN1) and connective tissue growth factor (CCN2): immediate-early gene products expressed in atherosclerotic lesions. Blood.

[CR282] Schotte D, Chau JC, Sylvester G, Liu G, Chen C, van der Velden VH, Broekhuis MJ, Peters TC, Pieters R, den Boer ML (2009). Identification of new microRNA genes and aberrant microRNA profiles in childhood acute lymphoblastic leukemia. Leukemia.

[CR283] Schutze N, Noth U, Schneidereit J, Hendrich C, Jakob F (2005). Differential expression of CCN-family members in primary human bone marrow-derived mesenchymal stem cells during osteogenic, chondrogenic and adipogenic differentiation. Cell Commun Signal.

[CR284] Segarini PR, Nesbitt JE, Li D, Hays LG, Yates JR, Carmichael DF (2001). The low density lipoprotein receptor-related protein/alpha2-macroglobulin receptor is a receptor for connective tissue growth factor. J Biol Chem.

[CR285] Shimo T, Nakanishi T, Kimura Y, Nishida T, Ishizeki K, Matsumura T, Takigawa M (1998). Inhibition of endogenous expression of connective tissue growth factor by its antisense oligonucleotide and antisense RNA suppresses proliferation and migration of vascular endothelial cells. J Biochem.

[CR286] Shimo T, Nakanishis T, Nishida T, Asano M, Kanyama M, Kuboki T, Tamatani T, Tezuka K, Takemura M, Matsumura T, Takigawa M (1999). Connective tissue growth factor induces the proliferation, migration, and tube formation of vascular endothelial cells in vitro, and angiogenesis in vivo. J Biochem.

[CR287] Shimo T, Nakanishi T, Nishida T, Asano M, Sasaki A, Kanyama M, Kuboki T, Matsumura T, Takigawa M (2001). Involvement of CTGF, a hypertrophic chondrocyte-specific gene product, in tumor angiogenesis. Oncology.

[CR288] Shimo T, Kubota S, Kondo S, Nakanishi T, Sasaki A, Mese H, Matsumura T, Takigawa M (2001). Connective tissue growth factor as a major angiogenic agent that is induced by hypoxia in a human breast cancer cell line. Cancer Lett.

[CR289] Shinde A, Epperly MW, Cao S, Holt D, Goff J, Shields D, Franicola D, Wipf P, Wang H, Greenberger JS (2014). Improved hematopoiesis in GS-Nitroxide (JP4–039)-treated mouse long-term bone marrow cultures and radioresistance of derived bone marrow stromal cell lines. In Vivo.

[CR290] Shi-wen X, Pennington D, Holmes A, Leask A, Bradham D, Beauchamp JR, Fonseca C, du Bois RM, Martin GR, Black CM, Abraham DJ (2000). Autocrine overexpression of CTGF maintains fibrosis: RDA analysis of fibrosis genes in systemic sclerosis. Exp Cell Res.

[CR291] Shi-Wen X, Renzoni EA, Kennedy L, Howat S, Chen Y, Pearson JD, Bou-Gharios G, Dashwood MR, du Bois RM, Black CM, Denton CP, Abraham DJ, Leask A (2007). Endogenous endothelin-1 signaling contributes to type I collagen and CCN2 overexpression in fibrotic fibroblasts. Matrix Biol.

[CR292] Sipkins DA, Wei X, Wu JW, Runnels JM, Cote D, Means TK, Luster AD, Scadden DT, Lin CP (2005). vivo imaging of specialized bone marrow endothelial microdomains for tumour engraftment. Nature.

[CR293] Sisco M, Kryger ZB, O'Shaughnessy KD, Kim PS, Schultz GS, Ding XZ, Roy NK, Dean NM, Mustoe TA (2008). Antisense inhibition of connective tissue growth factor (CTGF/CCN2) mRNA limits hypertrophic scarring without affecting wound healing in vivo. Wound Repair Regen.

[CR294] Smerdel-Ramoya A, Zanotti S, Stadmeyer L, Durant D, Canalis E (2008). Skeletal overexpression of connective tissue growth factor impairs bone formation and causes osteopenia. Endocrinology.

[CR295] Song Y, Lin Q, Cai Z, Hao T, Zhang Y, Zhu X (2019). Cysteine-rich protein 61 regulates the chemosensitivity of chronic myeloid leukemia to imatinib mesylate through the nuclear factor kappa B/Bcl-2 pathway. Cancer Sci.

[CR296] Su Y, Nishimoto T, Hoffman S, Nguyen XX, Pilewski JM, Feghali-Bostwick C (2019). Insulin-like growth factor binding protein-4 exerts antifibrotic activity by reducing levels of connective tissue growth factor and the C-X-C chemokine receptor 4. FASEB Bioadv.

[CR297] Sugiyama T, Kohara H, Noda M, Nagasawa T (2006). Maintenance of the hematopoietic stem cell pool by CXCL12-CXCR4 chemokine signaling in bone marrow stromal cell niches. Immunity.

[CR298] Sumiyoshi K, Kubota S, Furuta RA, Yasui K, Aoyama E, Kawaki H, Kawata K, Ohgawara T, Yamashiro T, Takigawa M (2010). Thrombopoietic-mesenchymal interaction that may facilitate both endochondral ossification and platelet maturation via CCN2. J Cell Commun Signal.

[CR299] Sun K, Wang Q, Huang XH (2006). PPAR gamma inhibits growth of rat hepatic stellate cells and TGF beta-induced connective tissue growth factor expression. Acta Pharmacol Sin.

[CR300] Sun D, Han S, Liu C, Zhou R, Sun W, Zhang Z, Qu J (2016). Microrna-199a-5p functions as a tumor suppressor via suppressing connective tissue growth factor (CTGF) in follicular thyroid carcinoma. Med Sci Monit.

[CR301] Sung DK, Kong WH, Park K, Kim JH, Kim MY, Kim H, Hahn SK (2013). Noncovalenly PEGylated CTGF siRNA/PDMAEMA complex for pulmonary treatment of bleomycin-induced lung fibrosis. Biomaterials.

[CR302] Suresh S, McCallum L, Lu W, Lazar N, Perbal B, Irvine AE (2011). MicroRNAs 130a/b are regulated by BCR-ABL and downregulate expression of CCN3 in CML. J Cell Commun Signal.

[CR303] Suresh S, McCallum L, Crawford LJ, Lu WH, Sharpe DJ, Irvine AE (2013). The matricellular protein CCN3 regulates NOTCH1 signalling in chronic myeloid leukaemia. J Pathol.

[CR304] Surmann-Schmitt C, Sasaki T, Hattori T, Eitzinger N, Schett G, von der Mark K, Stock M (2012). The Wnt antagonist Wif-1 interacts with CTGF and inhibits CTGF activity. J Cell Physiol.

[CR305] Suzuma K, Naruse K, Suzuma I, Takahara N, Ueki K, Aiello LP, King GL (2000). Vascular endothelial growth factor induces expression of connective tissue growth factor via KDR, Flt1, and phosphatidylinositol 3-kinase-akt-dependent pathways in retinal vascular cells. J Biol Chem.

[CR306] Tabe Y, Konopleva M, Munsell MF, Marini FC, Zompetta C, McQueen T, Tsao T, Zhao S, Pierce S, Igari J, Estey EH, Andreeff M (2004). PML-RARalpha is associated with leptin-receptor induction: the role of mesenchymal stem cell-derived adipocytes in APL cell survival. Blood.

[CR307] Taichman RS, Emerson SG (1994). Human osteoblasts support hematopoiesis through the production of granulocyte colony-stimulating factor. J Exp Med.

[CR308] Takigawa M (2013). CCN2: a master regulator of the genesis of bone and cartilage. J Cell Commun Signal.

[CR309] Takigawa M (2018). An early history of CCN2/CTGF research: the road to CCN2 via hcs24, ctgf, ecogenin, and regenerin. J Cell Commun Signal.

[CR310] Tam EM, Morrison CJ, Wu YI, Stack MS, Overall CM (2004). Membrane protease proteomics: Isotope-coded affinity tag MS identification of undescribed MT1-matrix metalloproteinase substrates. Proc Natl Acad Sci U S A.

[CR311] Tesfai Y, Ford J, Carter KW, Firth MJ, O'Leary RA, Gottardo NG, Cole C, Kees UR (2012). Interactions between acute lymphoblastic leukemia and bone marrow stromal cells influence response to therapy. Leuk Res.

[CR312] Tikellis C, Cooper ME, Twigg SM, Burns WC, Tolcos M (2004). Connective tissue growth factor is up-regulated in the diabetic retina: amelioration by angiotensin-converting enzyme inhibition. Endocrinology.

[CR313] Tong ZY, Brigstock DR (2006). Intrinsic biological activity of the thrombospondin structural homology repeat in connective tissue growth factor. J Endocrinol.

[CR314] Tsai KD, Chen W, Wang SH, Hsiao YW, Chi JY, Wu HY, Lee YJ, Wong HY, Tseng MJ, Lin TH (2014). Downregulation of connective tissue growth factor by LPS/IFN-gamma-induced nitric oxide is reversed by aristolochic acid treatment in glomerular mesangial cells via STAT-1alpha and NF-kappaB signaling. Chem Biol Interact.

[CR315] Tsang M, Quesnel K, Vincent K, Hutchenreuther J, Postovit LM, Leask A (2020). Insights into Fibroblast Plasticity: Cellular Communication Network 2 Is Required for Activation of Cancer-Associated Fibroblasts in a Murine Model of Melanoma. Am J Pathol.

[CR316] Twigg SM, Chen MM, Joly AH, Chakrapani SD, Tsubaki J, Kim HS, Oh Y, Rosenfeld RG (2001). Advanced glycosylation end products up-regulate connective tissue growth factor (insulin-like growth factor-binding protein-related protein 2) in human fibroblasts: a potential mechanism for expansion of extracellular matrix in diabetes mellitus. Endocrinology.

[CR317] Valle-Tenney R, Rebolledo DL, Lipson KE, Brandan E (2020). Role of hypoxia in skeletal muscle fibrosis: Synergism between hypoxia and TGF-beta signaling upregulates CCN2/CTGF expression specifically in muscle fibers. Matrix Biol.

[CR318] Vatanmakanian M, Tavallaie M, Ghadami S (2019). Imatinib independent aberrant methylation of NOV/CCN3 in chronic myelogenous leukemia patients: a mechanism upstream of BCR-ABL1 function?. Cell Commun Signal.

[CR319] Ventura A, Young AG, Winslow MM, Lintault L, Meissner A, Erkeland SJ, Newman J, Bronson RT, Crowley D, Stone JR, Jaenisch R, Sharp PA, Jacks T (2008). Targeted deletion reveals essential and overlapping functions of the miR-17 through 92 family of miRNA clusters. Cell.

[CR320] Vial C, Gutierrez J, Santander C, Cabrera D, Brandan E (2011). Decorin interacts with connective tissue growth factor (CTGF)/CCN2 by LRR12 inhibiting its biological activity. J Biol Chem.

[CR321] Vorwerk P, Wex H, Hohmann B, Oh Y, Rosenfeld RG, Mittler U (2000). CTGF (IGFBP-rP2) is specifically expressed in malignant lymphoblasts of patients with acute lymphoblastic leukaemia (ALL). Br J Cancer.

[CR322] Vorwerk P, Wex H, Hohmann B, Mohnike K, Schmidt U, Mittler U (2002). Expression of components of the IGF signalling system in childhood acute lymphoblastic leukaemia. J Clin Pathol Mol Pathol.

[CR323] Wahab NA, Brinkman H, Mason RM (2001). Uptake and intracellular transport of the connective tissue growth factor: a potential mode of action. Biochem J.

[CR324] Wahab NA, Weston BS, Mason RM (2005). Connective tissue growth factor CCN2 interacts with and activates the tyrosine kinase receptor TrkA. J Am Soc Nephrol.

[CR325] Wang JJ, Ye F, Cheng LJ, Shi YJ, Bao J, Sun HQ, Wang W, Zhang P, Bu H (2009). Osteogenic differentiation of mesenchymal stem cells promoted by overexpression of connective tissue growth factor. J Zhejiang Univ Sci B.

[CR326] Wang X, McLennan SV, Allen TJ, Twigg SM (2010). Regulation of pro-inflammatory and pro-fibrotic factors by CCN2/CTGF in H9c2 cardiomyocytes. J Cell Commun Signal.

[CR327] Wei Q, Frenette PS (2018). Niches for hematopoietic stem cells and their progeny. Immunity.

[CR328] Welch MD, Greene WK, Kees UR (2013). Hypomethylation of the CTGF gene locus is a common feature of paediatric pre-B acute lymphoblastic leukaemia. Br J Haematol.

[CR329] Welch MD, Howlett M, Halse HM, Greene WK, Kees UR (2015). Novel CT domain-encoding splice forms of CTGF/CCN2 are expressed in B-lineage acute lymphoblastic leukaemia. Leuk Res.

[CR330] Wells JE, Howlett M, Cole CH, Kees UR (2015). Deregulated expression of connective tissue growth factor (CTGF/CCN2) is linked to poor outcome in human cancer. Int J Cancer.

[CR331] Wells JE, Howlett M, Halse HM, Heng J, Ford J, Cheung LC, Samuels AL, Crook M, Charles AK, Cole CH, Kees UR (2016). High expression of connective tissue growth factor accelerates dissemination of leukaemia. Oncogene.

[CR332] Wexler SA, Donaldson C, Denning-Kendall P, Rice C, Bradley B, Hows JM (2003). Adult bone marrow is a rich source of human mesenchymal ‘stem’ cells but umbilical cord and mobilized adult blood are not. Br J Haematol.

[CR333] Winkler IG, Barbier V, Nowlan B, Jacobsen RN, Forristal CE, Patton JT, Magnani JL, Levesque JP (2012). Vascular niche E-selectin regulates hematopoietic stem cell dormancy, self renewal and chemoresistance. Nat Med.

[CR334] Wong M, Siegrist M, Goodwin K (2003). Cyclic tensile strain and cyclic hydrostatic pressure differentially regulate expression of hypertrophic markers in primary chondrocytes. Bone.

[CR335] Wunderlich K, Senn BC, Todesco L, Flammer J, Meyer P (2000). Regulation of connective tissue growth factor gene expression in retinal vascular endothelial cells by angiogenic growth factors. Graefe's Arch Clin Exp Ophtalmol.

[CR336] Xiao C, Srinivasan L, Calado DP, Patterson HC, Zhang B, Wang J, Henderson JM, Kutok JL, Rajewsky K (2008). Lymphoproliferative disease and autoimmunity in mice with increased miR-17–92 expression in lymphocytes. Nat Immunol.

[CR337] Xu J, Smock SL, Safadi FF, Rosenzweig AB, Odgren PR, Marks SC, Owen TA, Popoff SN (2000). Cloning the full-length cDNA for rat connective tissue growth factor: implications for skeletal development. J Cell Biochem.

[CR338] Yamaai T, Nakanishi T, Asano M, Nawachi K, Yoshimichi G, Ohyama K, Komori T, Sugimoto T, Takigawa M (2005). Gene expression of connective tissue growth factor (CTGF/CCN2) in calcifying tissues of normal and cbfa1-null mutant mice in late stage of embryonic development. J Bone Miner Metab.

[CR339] Yamashiro T, Fukunaga T, Kobashi N, Kamioka H, Nakanishi T, Takigawa M, Takano-Yamamoto T (2001). Mechanical stimulation induces CTGF expression in rat osteocytes. J Dent Res.

[CR340] Yan LF, Wei YN, Nan HY, Yin Q, Qin Y, Zhao X, Chen BY, Zhao G, Wei JG, Cui GB (2014). Proliferative phenotype of pulmonary microvascular endothelial cells plays a critical role in the overexpression of CTGF in the bleomycin-injured rat. Exp Toxicol Pathol.

[CR341] Yang DH, Kim HS, Wilson EM, Rosenfeld RG, Oh Y (1998). Identification of glycosylated 38-kDa connective tissue growth factor (IGFBP-related protein 2) and proteolytic fragments in human biological fluids, and up-regulation of IGFBP-rP2 expression by TGF-beta in Hs578T human breast cancer cells. J Clin Endocrinol Metab.

[CR342] Yang M, Huang H, Li J, Li D, Wang H (2004). Tyrosine phosphorylation of the LDL receptor-related protein (LRP) and activation of the ERK pathway are required for connective tissue growth factor to potentiate myofibroblast differentiation. FASEB J.

[CR343] Yeger H, Perbal B (2016). CCN family of proteins: critical modulators of the tumor cell microenvironment. J Cell Commun Signal.

[CR344] Yeh CH, Moles R, Nicot C (2016). Clinical significance of microRNAs in chronic and acute human leukemia. Mol Cancer.

[CR345] Yendamuri S, Calin GA (2009). The role of microRNA in human leukemia: a review. Leukemia.

[CR346] Yokoi H, Mukoyama M, Nagae T, Mori K, Suganami T, Sawai K, Yoshioka T, Koshikawa M, Nishida T, Takigawa M, Sugawara A, Nakao K (2004). Reduction in connective tissue growth factor by antisense treatment ameliorates renal tubulointerstitial fibrosis. J Am Soc Nephrol.

[CR347] Yoon PO, Park JW, Lee CM, Kim SH, Kim HN, Ko Y, Bae SJ, Yun S, Park JH, Kwon T, Kim WS, Lee J, Lu Q, Kang HR, Cho WK, Elias JA, Yang JS, Park HO, Lee K, Lee CG (2016). Self-assembled micelle interfering RNA for effective and safe targeting of dysregulated genes in pulmonary fibrosis. J Biol Chem.

[CR348] Yoshihara H, Arai F, Hosokawa K, Hagiwara T, Takubo K, Nakamura Y, Gomei Y, Iwasaki H, Matsuoka S, Miyamoto K, Miyazaki H, Takahashi T, Suda T (2007). Thrombopoietin/MPL signaling regulates hematopoietic stem cell quiescence and interaction with the osteoblastic niche. Cell Stem Cell.

[CR349] Yosimichi G, Kubota S, Nishida T, Kondo S, Yanagita T, Nakao K, Takano-Yamamoto T, Takigawa M (2006). Roles of PKC, PI3K and JNK in multiple transduction of CCN2/CTGF signals in chondrocytes. Bone.

[CR350] Zhang X, Chen X, Liu J, Dong X, Jin Y, Tian Y, Xue Y, Chen L, Chang Y, Liu Y, Wang J (2015). Knockdown of WISP1 inhibit proliferation and induce apoptosis in ALL Jurkat cells. Int J Clin Exp Pathol.

[CR351] Zhang X, Wang Y, Guo Q, Diao Y, Liu H, Song G, Wang W, Zhang Z, Yin H, Li L (2018). Prognostic role of microRNA-155 in patients with leukemia: a meta-analysis. Clin Chim Acta.

[CR352] Zhao C, Chen W, Yang L, Stimpson SA, Diehl AM (2006). PPARgamma agonists prevent TGFbeta1/Smad3-signaling in human hepatic stellate cells. Biochem Biophys Res Commun.

[CR353] Zhao M, Tao F, Venkatraman A, Li Z, Smith SE, Unruh J, Chen S, Ward C, Qian P, Perry JM, Marshall H, Wang J, He XC, Li L (2019). N-cadherin-expressing bone and marrow stromal progenitor cells maintain reserve hematopoietic stem cells. Cell Rep.

[CR354] Zhou BO, Yu H, Yue R, Zhao Z, Rios JJ, Naveiras O, Morrison SJ (2017). Bone marrow adipocytes promote the regeneration of stem cells and haematopoiesis by secreting SCF. Nat Cell Biol.

[CR355] Zhu RJ, Wu MQ, Li ZJ, Zhang Y, Liu KY (2013). Hematopoietic recovery following chemotherapy is improved by BADGE-induced inhibition of adipogenesis. Int J Hematol.

[CR356] Zhu X, Song Y, Wu C, Pan C, Lu P, Wang M, Zheng P, Huo R, Zhang C, Li W, Lin Y, Cao Y, Li N (2016). Cyr61 participates in the pathogenesis of acute lymphoblastic leukemia by enhancing cellular survival via the AKT/NF-kappaB signaling pathway. Sci Rep.

